# Tantalum and magnesium nanoparticles enhance the biomimetic properties and osteo-angiogenic effects of PCL membranes

**DOI:** 10.3389/fbioe.2022.1038250

**Published:** 2022-11-24

**Authors:** Jiangyu Nan, Wenbin Liu, Kai Zhang, Yan Sun, Yihe Hu, Pengfei Lei

**Affiliations:** ^1^ Department of Orthopedic Surgery, National Clinical Research Center for Geriatric Disorders, Xiangya Hospital, Central South University, Changsha, China; ^2^ Hunan Engineering Research Center of Biomedical Metal and Ceramic Implants, Changsha, China; ^3^ Department of Orthopedics, The First Affiliated Hospital, Medical College of Zhejiang University, Hangzhou, China

**Keywords:** periosteum, electrospinning, tantalum, magnesium, angiogenesis

## Abstract

Segmental bone defects, accompanied by periosteum stripping or injury, usually lead to delayed bone union or nonunion, which have challenged orthopedic surgeons. The periosteum, which provides essential blood supply and initial stem cells for bone tissue, plays an important role in the repair of bone defects. The reconstruction of the destroyed periosteum has attracted the attention of researchers exploring more satisfactory therapies to repair bone defects. However, periosteum-like biomaterials have yet to meet the clinical requirements and resolve this challenging problem. In this study, we manufactured a nanofiber periosteum replacement based on poly-ε-caprolactone (PCL), in which tantalum nanoparticles (TaNPs) and nanoscale magnesium oxide (MgO) were introduced to enhance its osteogenic and angiogenic ability. The results of *in vitro* experiments indicated that the PCL/Ta/MgO periosteum replacement, with excellent cytocompatibility, promoted the proliferation of both bone marrow mesenchymal stem cells (BMSCs) and endothelial progenitor cells (EPCs). Furthermore, the incorporation of TaNPs and nano-MgO synergistically enhanced the osteogenic differentiation of BMSCs and the angiogenic properties of EPCs. Similarly, the results of *in vivo* experiments from subcutaneous implantation and critical-sized calvarial defect models showed that the PCL/Ta/MgO periosteum replacement combined the osteogenesis and angiogenesis abilities, promoting vascularized bone formation to repair critical-sized calvarial defects. The results of our study suggest that the strategy of stimulating repairing bone defects can be achieved with the periosteum repaired *in situ* and that the proposed periosteum replacement can act as a bioactive medium to accelerate bone healing.

## Introduction

Bone defects are not uncommon in orthopedic clinical practice and can be caused by high-energy fractures, tumor destruction, or serious bone infection. The repair of bone defects remains a major challenge for orthopedic clinicians because of the apparent deficiencies of current therapies (Calori et al., 2011). Research on repairing bone defects has focused more on implant materials, structures, and drug delivery (Zeng et al., 2019; Park et al., 2021), while overlooking the importance of periosteum repair (Ho-Shui-Ling et al., 2018). Covering the bone surface, the periosteum comprises several functional layers in which the inner layer, or the inner cambium layer in another taxonomy, contains cells with osteogenic potential (Allen et al., 2004; Dwek, 2010). The periosteum is a highly microvascularized connective tissue with an abundant capillary system that can provide necessary substances and cells for osteogenesis and also plays a vital role in the repair of bone defects (Orwoll et al., 2003; Gao et al., 2019). Numerous studies have demonstrated the remarkable osteogenic potential of the periosteum; thus, constructing biomimetic periosteum replacement for bone defect repair has increasingly attracted the attention of researchers.

Several studies have investigated various biological and polymeric materials to construct periosteum replacements, including decellularized periosteum (Chen et al., 2015), periosteal extracellular matrix (PEM) hydrogel (Qiu P. et al., 2020), collagen type I (Col-I) (Khorsand et al., 2019), chitosan (Bombaldi de Souza et al., 2020), silk fibroin (Shuai et al., 2021), polylactic-co-glycolic acid (PLGA) nanosheets (Shi et al., 2014), poly-l-lactic acid (PLLA) (Lu et al., 2020), and poly-ε-caprolactone (PCL). Among these, PCL has been confirmed as a potential candidate to prepare a unique mimetic periosteum replacement because of its excellent elasticity and processability, as well as good biocompatibility and moderate degradation *in vivo* (Felice et al., 2018; Ji et al., 2019). However, despite its biocompatibility, PCL has poor effects on osteogenesis and angiogenesis (Toffoli et al., 2020). Therefore, enhancing the bioactivity of PCL to realize the bionic function of the periosteum replacement is currently the main research focus.

Tantalum (Ta) has been widely applied as an implant in both orthopedic and dental clinical practices in recent years. The corrosion resistance endowed by the outer oxide layer and excellent bioactivity compared to its inherent inertia makes Ta an excellent biomaterial for bone defect repair (Levine et al., 2006; Torstrick et al., 2016). Ta can be applied either as porous implants or nanoparticles, both of which exhibit great biocompatibility and osteogenic properties. Zhu et al. combined tantalum nanoparticles (TaNPs) and polyetheretherketone (PEEK) powder and fabricated a composite scaffold to repair bone defects. The TaNPs enhanced the mechanical properties and osteoinductivity of the PEEK material (Zhu et al., 2019). In our previous study, we blended TaNPs and PCL and found that 5 wt% of TaNPs showed the best effect on enhancing PCL crystallinity and hydrophilicity and further promoting its osteogenic properties (Xiong et al., 2021). Meanwhile, our team compared the influence of nano- and micro-scale tantalum particles in activating macrophages. The results of that study demonstrated that TaNPs were better than microparticles at attenuating the inflammatory microenvironment (Sun et al., 2022). These results demonstrated the potential of utilizing TaNPs to reinforce the mechanical properties and bioactivities of PCL to allow the PCL-based periosteum replacement to achieve the required osteogenic properties.

Periosteum or periosteum-like materials used to repair bone defects require both excellent osteogenic and angiogenic abilities. Extensive studies in recent years have proved the significant effects of magnesium oxide (MgO) on increasing the osteogenic activity of implants, boosting new bone formation, and facilitating osseointegration (Yuan et al., 2019; Yuan et al., 2020; Lin et al., 2021). More importantly, Mg^2+^ plays a pivotal role in facilitating angiogenesis stimulation of vascularized bone formation (Liu M. et al., 2022; Chen et al., 2022). Liu et al. manufactured PCL/gelatin electrospun membranes containing MgO nanoparticles, which showed improved proangiogenic activity both *in vitro* and *in vivo*; moreover, these membranes showed better performance in diabetic wound healing (Liu M. et al., 2022). Lai et al. used PLGA, *ß*-tricalcium phosphate (β-TCP), and magnesium powder to fabricate a novel porous scaffold for the repair of challenging bone defects and concluded that the integration of magnesium element into the scaffold promoted neovascularization-mediated new bone formation (Lai et al., 2019). In addition, Mg coating on titanium-based or tantalum-based scaffolds showed prominent osteogenic and angiogenic properties as well as increased early vascularization *in vivo* (Ma et al., 2020; Zhang C. N. et al., 2021)*.*

In this study, we prepared a PCL/Ta/MgO periosteum replacement by electrospinning PCL, 5% TaNPs, and 1% nano-MgO. *In vitro* experiments were performed to assess the ability of the incorporated TaNPs and nano-MgO to promote the osteogenic differentiation of BMSCs and enhance the angiogenic properties of EPCs and evaluate the potential coupling effect on osteogenesis and angiogenesis. A subcutaneous implantation model was established to verify the angiogenic properties of the PCL/Ta/MgO periosteum replacement *in vivo* and a critical-sized calvarial defect was used to confirm that the periosteum replacement could promote periosteum regeneration and vascularized bone formation in the bone defect areas. Our findings demonstrate the great potential of this Ta and MgO nanoparticle-incorporated PCL-derived periosteum replacement for periosteal tissue regeneration.

## Materials and methods

### Preparation and characterization of MgO and electrospun fiber membranes

#### Preparation of electrospun membranes

TaNPs were obtained from Dk Nanotechnology (Beijing Dk Nano Technology Co., Ltd., China Size = 50 nm), while MgO nanoparticles were purchased from Macklin Biochemical (Shanghai Macklin Biochemical Co., Ltd., China Size = 50 nm). The PCL/Ta/MgO nanofiber membranes were prepared by electrospinning (Elite Series, Ucalery Co. Ltd. Beijing). Briefly, PCL was dissolved in 2,2,2-trifluoroethanol; TaNPs were then added to the dispersed PCL solution with a 5% (w/v) concentration according to our previous study. MgO nanoparticles were then added at 1% (w/v) concentration, integrating two studies from the team of Wu (Liu et al., 2020; Liu M. et al., 2022). The mixture was ultrasonicated for 30 m to obtain a homogeneous dispersion of the two nanoparticles. The solution was then loaded into a 10-ml injection syringe awaiting electrospinning. The contents of the syringe were flowed on the aluminum foil paper in the electric field between the needle tip (+5 kV) and aluminum foil (−2 kV) at 0.5 ml/h. Electrospun membranes made from pure PCL and PCL/Ta without MgO were also fabricated as control groups. The materials used in the biological experiments in this study were sterilized with ethylene oxide.

#### Characterization of the electrospun membranes

The characterization of the electrospun membranes prepared in this study included surface morphology, elemental composition, crystal structure, surface hydrophilicity, and mechanical properties. The 2D morphologies were observed by scanning electron microscopy (SEM, JEOL, JSM-IT200, Japan). The pore sizes of 100 random pores on the surface of the membranes were measured from the SEM images using ImageJ. The presence of elements in the membranes was detected by energy-dispersive X-ray spectroscopy (EDX). The crystalline phase structures of the nanofibers were determined by X-ray diffraction (XRD, D8 Advance, Bruker, Germany). The hydrophilicity of the nanofiber membranes was indicated by the water contact angle. One microliter of ultrapure water was added to the membrane, and the contact angle was measured using a contact angle test system (JC 2000C, Zhongchen, China). Stress–strain curves were drawn by stretching 4 cm × 1 cm rectangle membrane samples on a universal testing machine (Instron + MTS) at 5 mm/min to evaluate the mechanical properties of films doped with different nanoparticles.

### Ion release

The PCL/Ta/MgO membrane was cut into 1 cm × 1 cm rectangle samples and immersed in 5 ml ultrapure water in a centrifuge tube at 37°C. The centrifuge tubes were placed on a shaker for 14 days, and 1 ml ultrapure water was replaced in each tube on days 1, 3, 5, 7, 10, and 14. A single water sample was collected to test the Mg^2+^ concentration using inductively coupled plasma-optical emission spectroscopy (ICP-OES, Leeman Labs, United States).

### *In vitro* studies

#### Cell viability and proliferation

The *in vitro* osteogenic experiments were conducted using bone mesenchymal stem cells (BMSCs) extracted from the bone marrow of Sprague–Dawley (SD) rats as described previously (Liu W. B. et al., 2022). The *in vitro* angiogenic experiments were conducted using endothelial progenitor cells (EPCs) from our laboratory. BMSCs were cultured in F-12 medium (Gibco, United States) containing 10% (v/v) fetal bovine serum (FBS, Gibco, United States) and 1% (v/v) penicillin/streptomycin (P/S, Absin, China). The EPCs were cultured in M-199 medium (Gibco, United States). The cultures were placed in an incubator with 5% CO_2_ at 37°C. The BMSCs were passaged, and the cells were collected at passage 3 for further experiments.

A total of 1 × 10^4^ cells (BMSCs and EPCs) were seeded onto membranes to evaluate the biosafety of the different membranes. The viability of the two cell types on different membranes was assessed using a Calcein acetoxymethyl (Calcein AM) and propidium iodide (PI) double staining kit (Yeasen, China) after 24 h and 72 h of culture. The culture medium was removed, the membranes were washed with 1× assay buffer, and the prepared working solution was added to the samples. Living cells were labeled as green by Calcein-AM probe, while dead cells were simultaneously stained as red by the PI probe. The cells were observed using an inverted fluorescence microscope (Leica DMC6200, Germany).

Cell proliferation on the membranes was tested on days 1, 3, and 5 for BMSCs and days 1, 2, and 3 for EPCs. The cell-seeded membranes were incubated with 10% Cell Counting Kit-8 (CCK-8, NCM Biotech, C6005, Suzhou, China) solution prepared in complete medium for 4 h; 100 μL of the incubated solution was then transferred into a 96-well plate (Guangzhou Jet Bio-Filtration Co. Ltd., China). The absorbance of the samples was measured on a microplate reader (Multiskan GO, United States) at 450 nm.

#### Cell adhesion

The membranes were cropped into round samples 1 cm in diameter, and samples from the different groups were plated on 48-well plates (Jet Biofil, China). The cells were then seeded at 5 × 10^3^ cells per membrane. After culturing for 3 days, FITC phalloidin and 4′,6-diamidino-2-phenylindole (DAPI) staining was performed to stain the nuclei and F-actin of the cells, which were observed under a confocal laser scanning microscope (CLSM, Leica TCS SP8 X, Germany). After removing the culture media, the samples were washed with phosphate-buffered solution (PBS) before fixing the cells with a 4% paraformaldehyde (PFA) solution. Triton X-100 solution (0.5%) was used to permeabilize cells for 10 min, and the samples were washed again with PBS. FITC phalloidin (Yeasen, China) was diluted with 1% bovine serum albumin (BSA) solution at a ratio of 1:200. The solution was then added to the cells and incubated at room temperature for 1 h. After three rounds of washing for 5 min, 100× diluted DAPI (Yeasen, China) solution was added to the samples for nuclear staining and incubated for 10 min. After cleaning the residual dye with PBS, the membranes were transferred to confocal dishes for observation.

#### Alkaline phosphatase assay

To detect the expression of alkaline phosphatase (ALP) of BMSCs seeded on the membranes after inducing for 7 days, an ALP assay kit (Nanjing Jiancheng, China) was first used to measure ALP activity; a color development kit (Beyotime Biotechnology, China) was then used to stain ALP in the cells. A total of 1 × 10^4^ BMSCs were seeded onto the surface of the membrane samples in 48-well plates cultured in osteogenic medium. According to the manufacturer’s instructions, 5 μL of the supernatant from each well was transferred into a 96-well plate, followed by the successive addition of 50 μL of substrate solution and 50 μL of buffer. After incubating for 15 min at 37°C, color developer was added. Finally, the optical density at 520 nm was measured on a microplate reader (Multiskan GO, United States). After removing all medium from the wells, the samples were fixed with PFA, followed by the addition of the prepared staining solution. The results were observed and captured using an industrial camera.

#### Collagen staining

Collagen deposition in the BMSCs was detected by Sirius red staining. One milliliter of cell suspension containing 5 × 10^4^ cells was added to the membranes of different groups, using osteoblast-inducing conditioned media to culture cells, and the media was replaced every 3 d. After induction for 7 and 14 days, the cells on the membranes were fixed with 4% PFA, and picrosirius red staining solution (Phygene, China) was added for 18 h to stain collagen. The staining solution was washed off with 0.1 M acetic acid. A light microscope (Leica DMRBE, Germany) was used to obtain detailed images. To obtain semi-quantitative data, an eluent was prepared with 0.2 M NaOH and methanol at a ratio of 1:1 to elute the stained collagen. The absorbance was measured at a wavelength of 540 nm.

#### Mineralized nodule staining

Mineralized nodule formation from calcium deposition is a more intuitive representation of the osteogenic ability of cells growing on membranes. The PCL, PCL/Ta, and PCL/Ta/MgO membranes were cocultured with BMSCs in a 48-well plate for 1 d. The medium was then replaced with osteoblast-inducing conditioned media (αMEM medium supplemented with 10% FBS, 1% P/S, 10 nM dexamethasone, 50 μM ascorbic acid, and 10 mM *ß*-glycerin phosphate), which was replaced every 3 d. Alizarin red staining (ARS, Servicebio, China) was utilized to visualize the mineralized nodules on days 14 and 21 after induction, and images were captured as well. Ten percent cetylpyridinium chloride (Sinopharm Chemical Reagent Co., Ltd., China) solution was prepared to wash the stained nodules. The absorbance was detected at 562 nm.

#### *In vitro* vascularization assay

To evaluate the ability of the different nanofiber membranes to promote angiogenesis, cell tube formation assays were conducted. Matrigel (Corning Inc., NY, United States) was melted and used at 4°C. Matrigel (100 μL) was added to the wells of a 48-well plate to induce tube formation. The plate was placed at 4°C to ensure that the Matrigel in the wells was completely flat. When the gel solidified at 37°C, 5 ×10^4^ EPCs were seeded on the Matrigel and cultured with leach liquor of different membranes. The EPCs on Matrigel were observed after 4 h and photographed using an optical microscope. The photographs were then analyzed in ImageJ to count the total length, number, and number of branches of tube formation in five random fields.

#### Cell migration assay

Cell migration to the nanofiber membranes was assessed by Transwell migration assay. EPCs were collected and suspended in a complete medium at 10^5^ cells/mL. Cropped samples of the different membranes were spread out at the bottom of the lower chamber of the Transwell chamber (NEST Biotechnology, 701001, Wuxi, China), to which 500 μL of complete medium containing the EPCs was added. The EPCs were seeded on the polycarbonate film in the upper chamber, and the same medium was added. PFA-fixed crystal violet staining was performed to visualize EPCs on the permeable film of the chamber after culturing for 24 h. After washing with PBS three times, the upper side of the film was wiped with a swab to remove cells that did not migrate, while cells migrating to the lower side of the film were photographed using a microscope.

#### Real-time polymerase chain reaction analysis

To study the osteogenic induction and angiogenic promotion of the membranes, BMSCs and EPCs were seeded on the membranes and cultured with the respective conditioned media. After 7 d of culture for BMSCs and 1 d for EPCs, total RNA was extracted with TRIzol reagent, and cDNA was synthesized using a PrimeScript RT Reagent Kit (Takara, China) according to the manufacturers’ instructions. Quantitative real-time polymerase chain reaction (qRT-PCR) was conducted to compare the expression levels of osteogenic and angiogenic genes among the different groups. The detected osteogenic genes included runt-related transcription factor 2 (Runx2), bone morphogenetic protein-2 (BMP-2), and ALP, while the angiogenic genes included angiopoietin-1 (Ang-1) and endothelial nitric oxide synthase (eNOS). The reactions were performed on a LightCycler 480 (Roche, Germany) instrument using FastStart Universal SYBR Green Master Mix (Roche, Germany) as follows: 5 min at 95°C for one cycle, 10 s at 95°C, and 30 s at 60°C. Using the housekeeping gene GAPDH as an internal control gene, the relative expression levels of each target gene were quantitatively analyzed using the 2^−ΔΔCT^ method. The primer sequences of each gene used for the PCR amplification are listed in Table 1.

**TABLE 1:** Primer sequences for the genes used in the qRT-PCR analysis.

Gene	Primer sequence (5′ to 3′)
GAPDH	F: ATG​GCT​ACA​GCA​ACA​GGG​T
	R: TTATGGGGTCTGGGATGG
Runx2	F: TCGGAAAGGGACGAGAG
	R: TTC​AAA​CGC​ATA​CCT​GCA​T
ALP	F: CCT​ACT​TGT​GTG​GCG​TGA​A
	R: GCAGGATGGACGTGACC
BMP-2	F: AACACAAACAGCGGAAGC
	R: AGCCAGGGGAAAAGGAC
Ang-1	F: AGCCTGCGTCCTCTGTT
	R: CGTGTACCTGGGGTCGT
eNOS	F: TACTCCAGGCTCCCGATG
	R: AAGGGCAGCAAACCACTC

### Subcutaneous implantation

To confirm the angiogenic ability *in vivo*, membranes in different groups were implanted subcutaneously between the skin and muscle on the backs of SD rats. All *in vivo* experiments in this study were approved by the Biomedical Research Ethics Committee of Central South University (license number: CSU-2022-0530). Twelve male SD rats weighing 200–250 g (LabAnimal Research Center, Hunan, China) were divided into three groups for implantation of different membranes. Four round samples 1 cm in diameter were prepared and implanted in the rats according to the groups. The rats were euthanized on Day 14 after implantation. The subcutaneous samples and skin were excised together and fixed with 4% PFA. The fixed specimens were then sliced and stained for histological analysis. The sections were stained with hematoxylin and eosin (H&E) and Masson’s trichome for general analysis and collagen evaluation, respectively. Selected slices were also subjected to anti-CD31-α- smooth muscle actin (α-SMA) immunofluorescent double labeling to directly observe neovascularization. The relative fluorescence intensities of CD31 were calculated using ImageJ software.

### Critical-sized calvarial defect repair

A critical bone defect model was established to verify the osteogenic potential of the materials *in vivo*. Three SD rats per group received critical bone defects. The rats used in this experiment were the same as those used in the subcutaneous implantation experiment. A longitudinal incision, approximately 2 cm in length, was made on the top of the head and the periosteum of the parietal bone was removed. A defect 4 mm in diameter was then made by trephine on either side of the coronal suture. The defects were covered with the different membranes according to group. The incision was then carefully sutured. The rats were sacrificed by carbon dioxide asphyxiation to obtain the skulls after 4 weeks. *In vivo* new bone formation induced by implants was observed by micro-CT (Skyscan 1172, Belgium). Newly formed bone volume (BV) and bone volume/total volume (BV/TV) were quantitatively analyzed using CT Analyser software (v1.17) in three samples from each group, and new bone-reconstructed 3D models were made using Mimics (v20.0) software. The specimens were then decalcified and sectioned to observe histological bone formation using H&E and Masson’s trichome staining. Immunohistochemical analysis of the specimen slices was performed for CD31 and OCN to further characterize new bone formation and neovascularization in the defect areas. Immunofluorescent staining for periostin was also performed to examine periosteum regeneration.

### Statistical analysis

Data were analyzed using GraphPad 9 (GraphPad Software, United States). The results are expressed as mean ± standard deviation. The results for three and more groups were tested by one-way analysis of variance. The confidence levels were set at 95% (p* < 0.05), 99% (p** <0.01), 99.9% (p*** <0.001), and 99.99% (p**** <0.0001).

## Results and discussion

### Characterization of nanofibrous membranes

**FIGURE 1:**
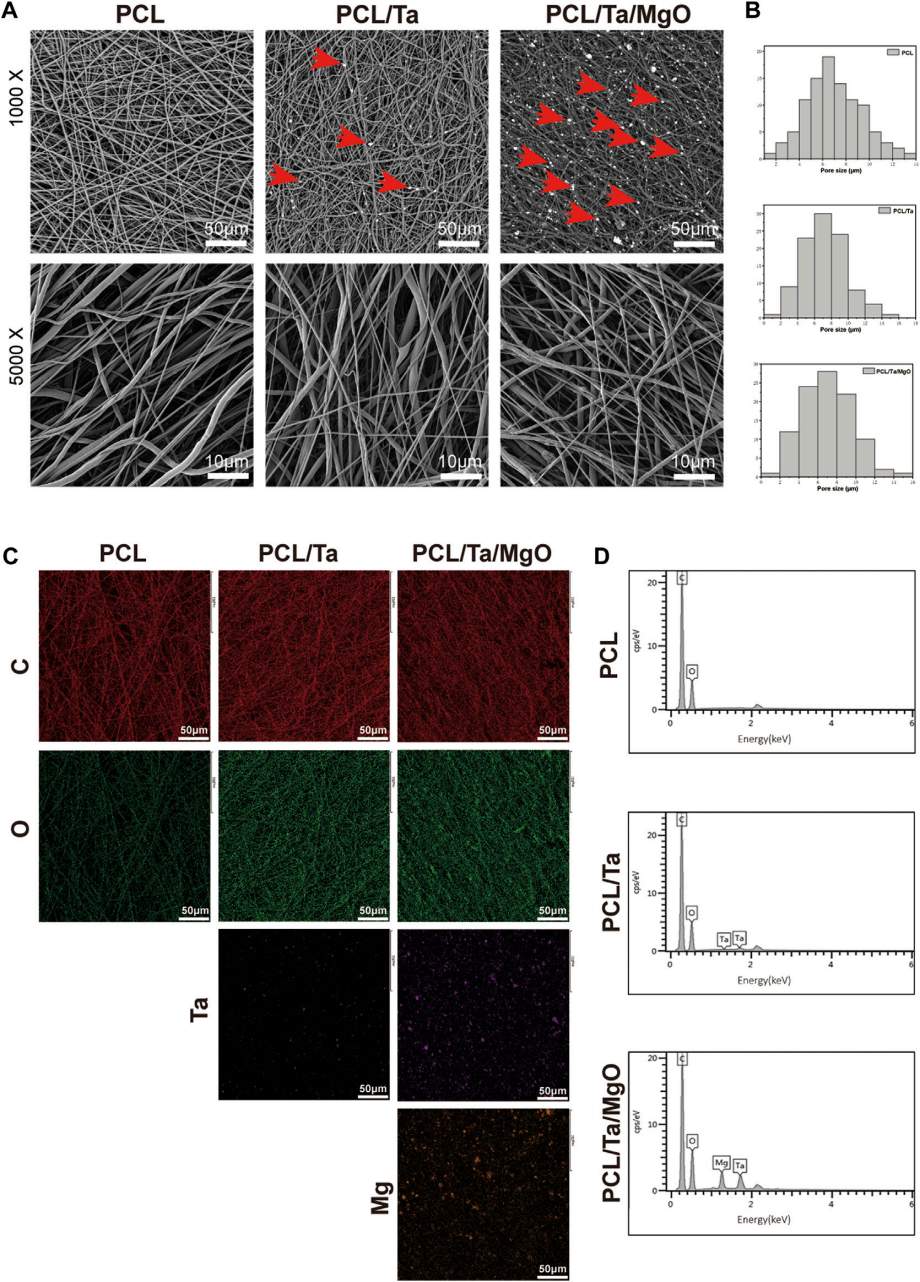
Morphological characterization and elemental analysis of different membranes. **(A)** SEM images at different magnifications. Red arrows: tantalum or magnesium oxide nanoparticles. **(B)** Distribution of pore sizes on the surface. (*n* = 100) **(C)** Element mapping. **(D)** EDS spectra.

This study successfully fabricated PCL, PCL/Ta, and PCL/Ta/MgO nanofiber membranes *via* electrospinning. The surface morphology of the nanofiber membranes was analyzed by SEM at different magnifications, which showed nanofibers with a smooth surface with irregular crosslinking, forming a porous structure on the surface, with pore sizes mainly ranging from 4 μm to 10 μm. (Figures 1A,B). Comparisons of SEM images at relatively low magnification showed brighter spots representing metal nanoparticles distributed randomly on the surface of the membranes. The high-magnification images showed nanofibers with irregular surfaces due to the random dispersion of TaNPs and nano-MgO inside the PCL matrix. EDS analysis of the elemental composition and capture element mapping of the membranes (Figures 1C,D) showed the incorporation of Ta and MgO into PCL, with uniform distribution of the two elements on the 2D surface.

**FIGURE 2:**
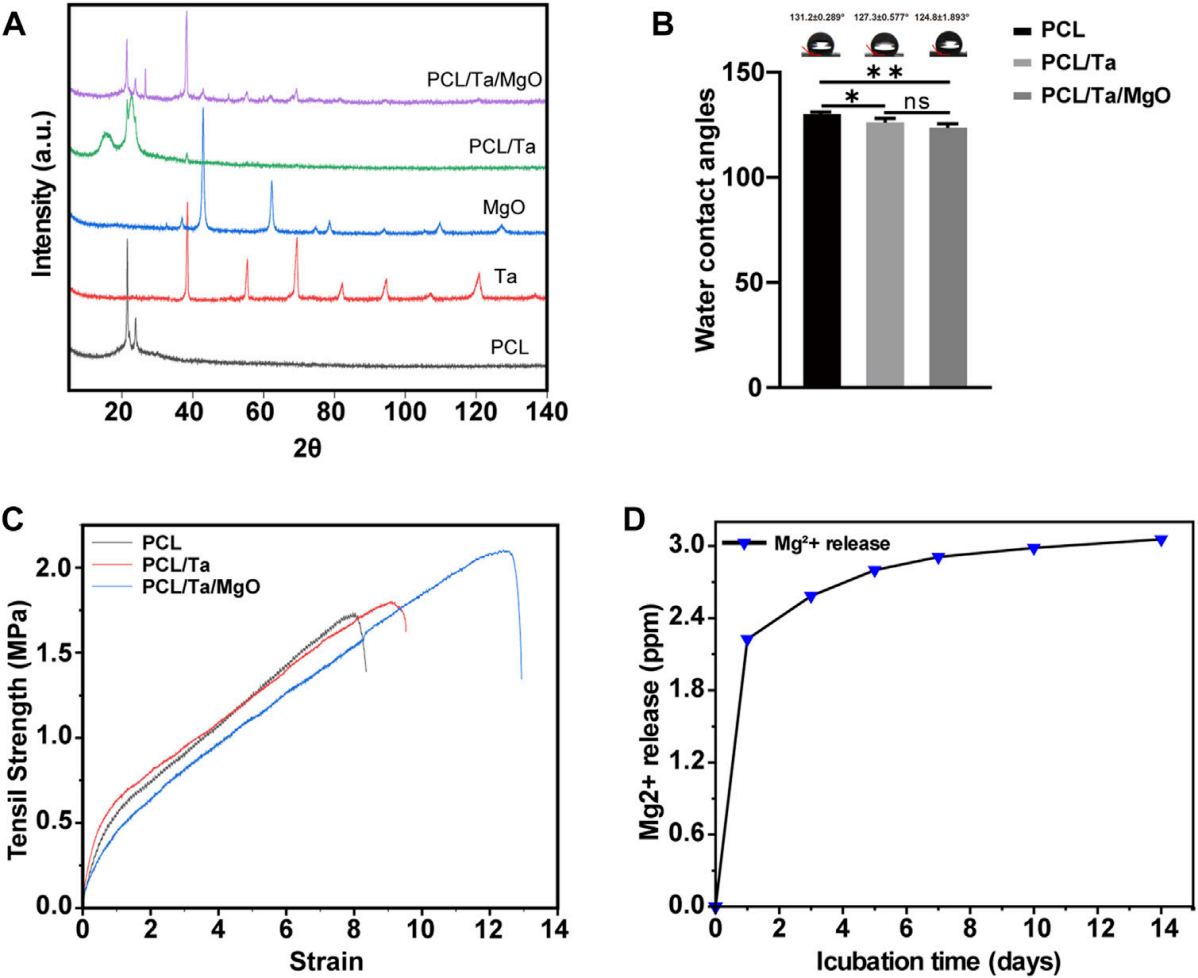
**(A)** XRD diffraction of PCL, tantalum nanoparticles, magnesium oxide nanoparticles, and nanocomposite membranes. **(B)** Water contact angles of PCL, PCL/Ta, and PCL/Ta/MgO membranes. Compared to pure PCL, PCL/Ta and PCL/Ta/MgO showed decreased contact angles (*n* = 3). **(C)** Representative stress–strain curves of the nanocomposite membranes. **(D)** Magnesium ion release from the PCL/Ta/MgO membrane. (*p < 0.05, **p < 0.01, ***p < 0.001, and ****p < 0.0001).

The specificity of the crystalline structure was detected by XRD. As shown in Figure 2A, the characteristic diffraction peaks of the TaNPs and nano-MgO were observed at 2θ = 38.24° and 42.88°, respectively, on the diffraction curve. The diffraction peaks at 2θ = 21.42° and 23.84° were attributed to PCL and were observed in all three nanofibers. The TaNPs and nano-MgO peaks in the membranes indicated their successful integration in the PCL matrix. The results confirmed that the actual amount of incorporation was close to the predesigned values.

The hydrophilicity of a biomaterial significantly affects cell attachment and subsequent cellular behavior. The modification of materials to enhance their bioactivity in many studies has focused on improving the hydrophilicity, which is evaluated by measuring the water contact angle. The contact angles of the nanofiber membranes in this study were 131.2 ± 0.289° for PCL, 127.3 ± 0.577° for PCL/Ta, and 124.8 ± 1.893°for PCL/Ta/MgO (Figure 2B). Some improvement in hydrophilicity compared to pure PCL was observed for the incorporation with TaNPs and nano-MgO; however, no significant difference was observed between PCL/Ta and PCL/Ta/MgO. The results revealed a favorable change in the hydrophilicity of the high-polymer material from the incorporation of Ta, as reported by Mei et al. (Mei et al., 2019) and Zhang et al. (Zhang X. et al., 2021) The incorporation of MgO did not improve the hydrophilicity.

Finally, the mechanical properties of the nanofiber membranes were tested using tensile-strength measurements. Analysis of the typical stress-strain curves of pure (Figure 2C) PCL, PCL/Ta, and PCL/Ta/MgO membrane showed the increased tensile strength of the PCL/Ta nanofiber membrane compared to pure PCL and a more significant increase for the membrane with both TaNPs and nano-MgO. Moreover, the elongation at the break of the membranes increased with the successive incorporation of the two nanoparticles. This favorable phenomenon may be attributed to the pinning effect of the Ta and nano-MgO dispersed in the PCL nanofibers.

### Ion release

Studies on biomaterials containing osteogenesis-related elements have reported that the local concentration of ions such as Ca, Mg, and P may positively affect the osteogenic induction of BMSCs (An et al., 2012; Zhai et al., 2013). Ta, which has extraordinarily stable chemical properties, seldom releases ions into its surrounding environment. Therefore, Mg^2+^ was the main ion released by the PCL/Ta/MgO membrane. To determine the ion release kinetics of the membrane containing MgO, the aqueous medium was collected at intermittent time points and measured by ICP-OES (Figure 2D). The results were organized into a line chart of the cumulative release of Mg^2+^. The amount of released Mg^2+^ on the first day reached 73% of the equilibrium release and increased to 85% on the third day. On the tenth day, the Mg^2+^ concentration in the aqueous medium reached an equilibrium, indicating a continuous release of Mg^2+^ from the membrane. In summary, the release of Mg^2+^ from the PCL/Ta/MgO membrane reached equilibrium within 14 days, creating a suitable environment for osteogenic and angiogenic differentiation (Lai et al., 2019; Zhang et al., 2019).

### Membrane cytocompatibility

**FIGURE 3:**
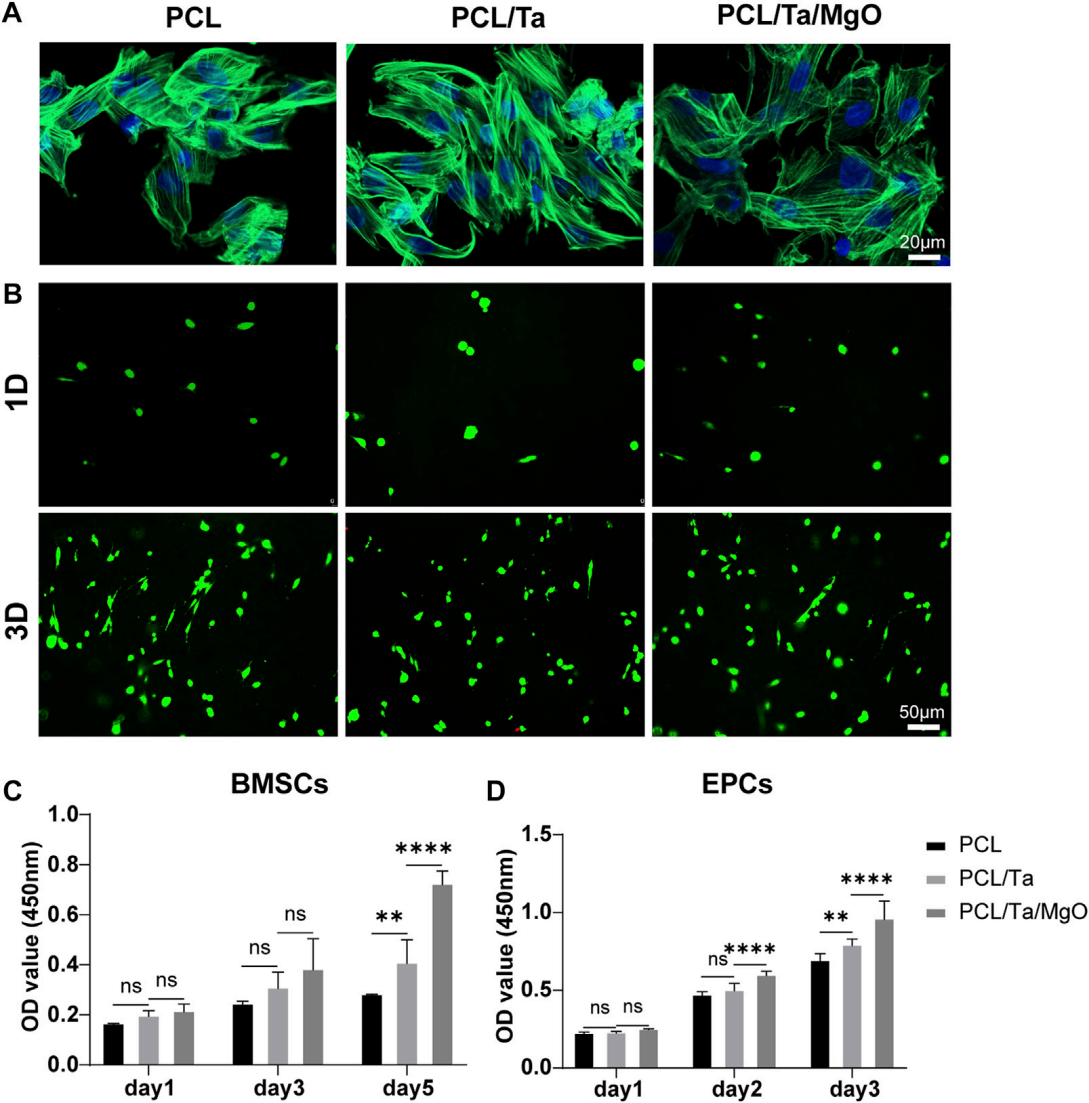
Cytocompatibility of PCL and different nanoparticle-doped membranes. **(A)** BMSC morphology on different membranes on day 1; **(B)** Live/dead staining of BMSCs cultured on the membranes after 1 d and 3 d; CCK-8 assay of **(C)** BMSC proliferation on days 1, 3, and 5 and **(D)** EPC proliferation on days 1, 2, and 3. (*n* = 6) (*p < 0.05, **p < 0.01, ***p < 0.001, and ****p < 0.0001).

The satisfactory repair of bone defects requires cells to spread and proliferate after their initial attachment to the biomaterials. The interaction between cells and biomaterials can further regulate the differentiation of seeded cells. In this study, we first assessed the effect of different electrospun membranes on the viability and proliferation of BMSCs and EPCs to guarantee subsequent osteogenic and angiogenic differentiation. BMSC cytoskeletons were outlined with phalloidin staining, in which BMSCs adhere and spread on the surfaces of the three membranes after 1 day of culture (Figure 3A). Cell viability was assessed by live/dead staining on days 1 and 3 after culturing, in which viable cells were stained green by Calcein-AM and dead cells were stained red by PI. Few dead BMSCs and EPCs were observed on the membranes, and the number of live cells increased steadily over time in each group. In addition, the number of living cells at the same time did not differ significantly among the three groups, which indicated that the incorporation of TaNPs or nano-MgO did not cause cytotoxicity to BMSCs (Figure 3B) or EPCs (Supplementary Figure S1A). The results were further confirmed by CCK-8 assays, in which cells proliferated on membranes containing different nanoparticles and pure PCL membranes showed no difference in BMSC proliferation on the first 3 days. However, the incorporation of metal nanoparticles promoted cell proliferation at day 5, possibly due to the potential bioactivity of TaNPs and nano-MgO (Figure 3C). A similar proliferation trend was observed in EPCs (Figure 3D). These results indicated the excellent cytocompatibility of these nanofiber membranes.

### *In vitro* osteogenic differentiation

**FIGURE 4:**
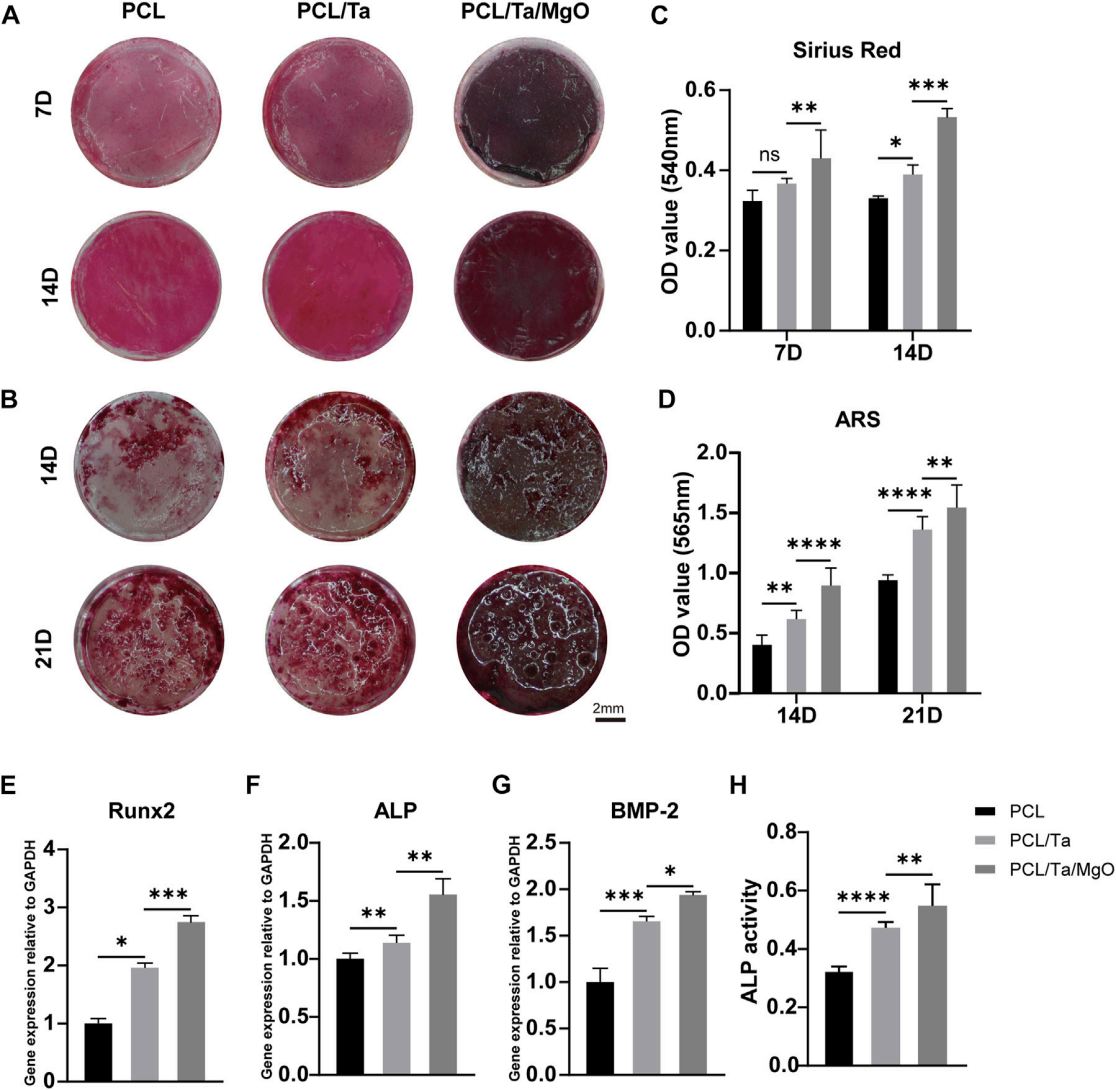
Evaluation of the *in vitro* osteogenic differentiation of BMSCs on different membranes. **(A)** Representative images of Sirius red staining on days 7 and 14 after osteogenic induction and **(B)** Alizarin red S (ARS) staining on days 14 and 21. Semi-quantitative analysis of **(C)** Sirius red and **(D)** ARS staining (*n* = 6). Gene expression levels of osteogenesis-related factors **(E)** Runx2, **(F)** ALP, and **(G)** BMP-2 genes in BMSCs on day 7 (*n* = 3). **(H)** ALP activity by BMSCs on different membranes. (*n* = 6) (*p < 0.05, **p < 0.01, ***p < 0.001, and ****p < 0.0001).

As a biomaterial aimed at repairing the functional periosteum, an excellent osteogenic ability is the basic required property of the periosteum replacement. Many bioactive metal materials with osteogenic properties serve as bone substitutes in the form of implants or as modifiers of other materials. TaNPs, with a larger specific surface area than that of Ta metal, also promote the osteogenic differentiation of stem cells (Park et al., 2019; Zhu et al., 2019). In the present study, Sirius red staining was used to detect type-I collagen (COL I) secreted by BMSCs as the main component of the extracellular matrix, the mineralization of which was then evaluated as a late biomarker of osteogenic differentiation using Alizarin red staining (ARS). To compare the ability to induce the osteogenesis of membranes containing TaNPs and nano-MgO, collagen deposition, mineralization, and ALP activity and expression were measured and RT-PCR was conducted to confirm the expression of genes associated with osteogenesis. More collagen was deposited in the presence of TaNPs, and the amount further increased when nano-MgO was also incorporated (Figure 4A). The optical density (OD) values measured from cell-seeded PCL/Ta/MgO membranes showed the highest collagen levels (Figure 4C). Calcium deposition on the three kinds of membranes was observed 14 and 21 days after inducing osteogenesis. The levels increased over time, with the PCL/Ta/MgO membrane showing the highest amount of calcium nodules (Figure 4B), consistent with the quantitative results (Figure 4D). The combination of the widely validated ability of Ta to promote osteogenesis and the possible osteogenic differentiation ability of MgO contributed to these results. The osteogenic gene expression levels of ALP, BMP-2, and Runx2 detected by RT-PCR were analyzed and illustrated in Figures 4E–G. The BMP-2/Smad/Runx2 signaling pathway plays a pivotal role in osteoblast differentiation and maturation (Zhang et al., 2020). Furthermore, the transcription factor Runx2, which is highly expressed in osteoblasts, binds to specific cis-acting elements and regulates the expression of bone-specific matrix proteins (COL I, OPN, OCN, and BSP), thus promoting bone formation (Franceschi et al., 2003; Qiu W. X. et al., 2020). ALP assays were performed to identify ALP on BMSCs as an early biomarker of osteogenic activity. Figure 4H shows that the incorporation of TaNPs in the membrane significantly increased ALP activity 7 days after osteogenic induction, with the PCL/Ta/MgO membrane group showing the highest ALP activity. The ALP staining density on the PCL/Ta/MgO membranes was also the highest compared to that of the other two membranes (Supplementary Figure S1B). These results indicated that compared to the pure PCL membrane, the bone formation-related gene expression levels increased in BMSCs growing on membranes modified with TaNPs or TaNPs/nano-MgO.

The ability of Ta and MgO to induce osteogenic differentiation and promote bone formation has been broadly confirmed in recent years. Kang et al. demonstrated that TaNP-induced autophagy in MC3T3-E1 osteoblasts promoted cell proliferation (Kang et al., 2017). In addition, a comprehensive review by Qian et al. concluded that Ta showed an inductive effect on bone formation by activating several probable osteogenesis-related signaling pathways (Qian et al., 2021). Similarly, Kim et al. suggested that Mg^2+^ released from materials can stimulate the ERK pathway of BMSCs to achieve a positive effect on inducing osteogenic differentiation (Kim et al., 2017). Our *in vitro* experiments on BMSCs verified the synergetic effect of TaNPs and nano-MgO in PCL/Ta/MgO periosteum replacement on enhancing bone formation, demonstrating its promise for the repair of periosteal defects.

### *In vitro* angiogenetic and cell migration assay

**FIGURE 5:**
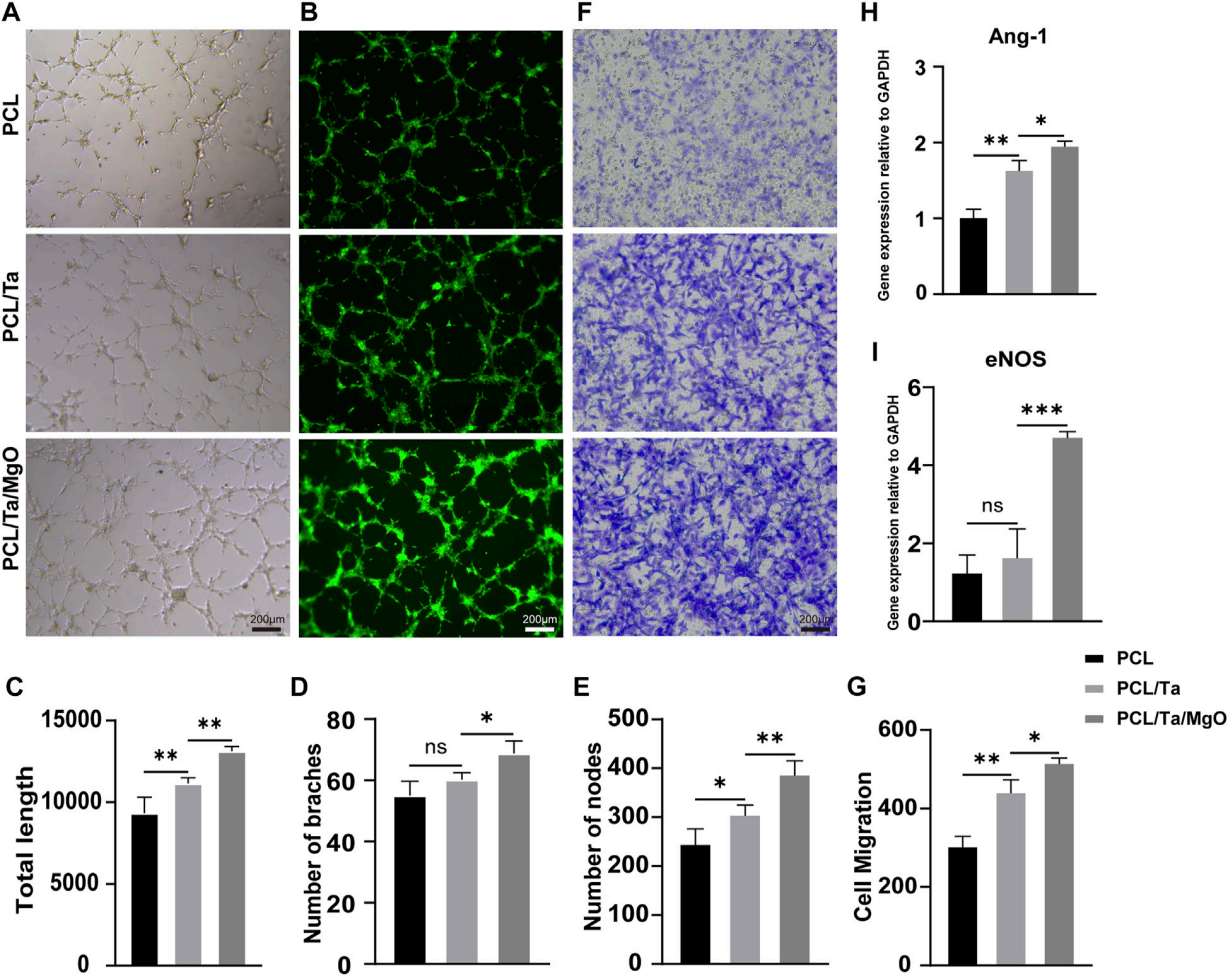
Evaluation of the *in vitro* angiogenic ability of EPCs on different membranes. **(A)** Representative bright-field images, **(B)** fluorescence images stained with Calcein-AM, and **(C–E)** quantitative analysis of the tube formation assay at 4 h. **(F)** Representative images and **(G)** quantitative analysis of the Transwell assay on day 1. Expression levels of angiogenesis-related **(H)** Ang-1 and **(I)** eNOS genes in EPCs on day 1 (*n* = 3). (*p < 0.05, **p < 0.01, ***p < 0.001, and ****p < 0.0001).

The periosteum covers the surface of cortical bone and is mostly supplied by the embedded vascular network. Biomimetic periosteum material should ideally not only induce osteogenesis but also facilitate the formation and sprouting of vessels *in situ* to provide synergistic effects during bone regeneration. The results of the *in vitro* tube formation assay showed different abilities of the three membranes to induce EPCs to form tubular structures. The real-time process of tube formation was observed, and bright-field (Figure 5A) and fluorescence (Figure 5B) images stained by Calcein-AM were captured. Quantification of tubular structures was performed in ImageJ, and we compared the total length (Figure 5C), number of branches (Figure 5D), and number of nodes (Figure 5E) among the different membranes. Our results indicated that the PCL/Ta and PCL/Ta/MgO membrane induced more tubule networks in the early stages than pure PCL. The incorporation of nano-MgO into the PCL/Ta membrane and the subsequent Mg^2+^ release seemed to induce the highest angiogenic ability. Transwell migration assays were used to evaluate the migration ability of EPCs cultured on diverse membranes (Figures 5F,G), in which cells that migrated across the chamber membranes were stained with crystal violet and the number of cells was counted in ImageJ. The PCL/Ta/MgO periosteum replacement promoted EPC migration to a higher extent than PCL and PCL/Ta when cultured in Transwell chambers for 24 h, which indicated its potential to recruit more local vascular endothelial cells to the implantation site when used for periosteum regeneration.

Additionally, RT-PCR was used to detect the expression levels of angiogenesis-related genes, including Ang-1 and eNOS. The results showed that the PCL/Ta/MgO membrane induced the highest expression of these genes (Figures 5H, I). Nitric oxide (NO) is one of the most important contributors to the angiogenic response (Chen et al., 2004), while eNOS expression stimulates endothelial cells to produce NO, which can partially stimulate Ang-1-induced angiogenesis.

The formation of new bone and reconstruction of the vascular network are the most essential steps in bone regeneration and can promote each other (Yang et al., 2021) to achieve a synergistic effect. Many studies on Mg-containing scaffolds have also reported “osteogenic–angiogenic” coupling effects, which showed excellent effects in repairing bone defects. Our periosteum replacement combined the strong osteoinductive abilities of Ta and MgO and the outstanding angiogenic inducibility of MgO; thus, we successfully developed an ideal periosteal material for periosteum regeneration. In this study, the incorporation of TaNPs in the nanofiber membranes significantly promoted EPC migration and tube formation; however, additional study is required regarding its specific mechanisms.

### *In vivo* osteogenesis and angiogenesis

**FIGURE 6:**
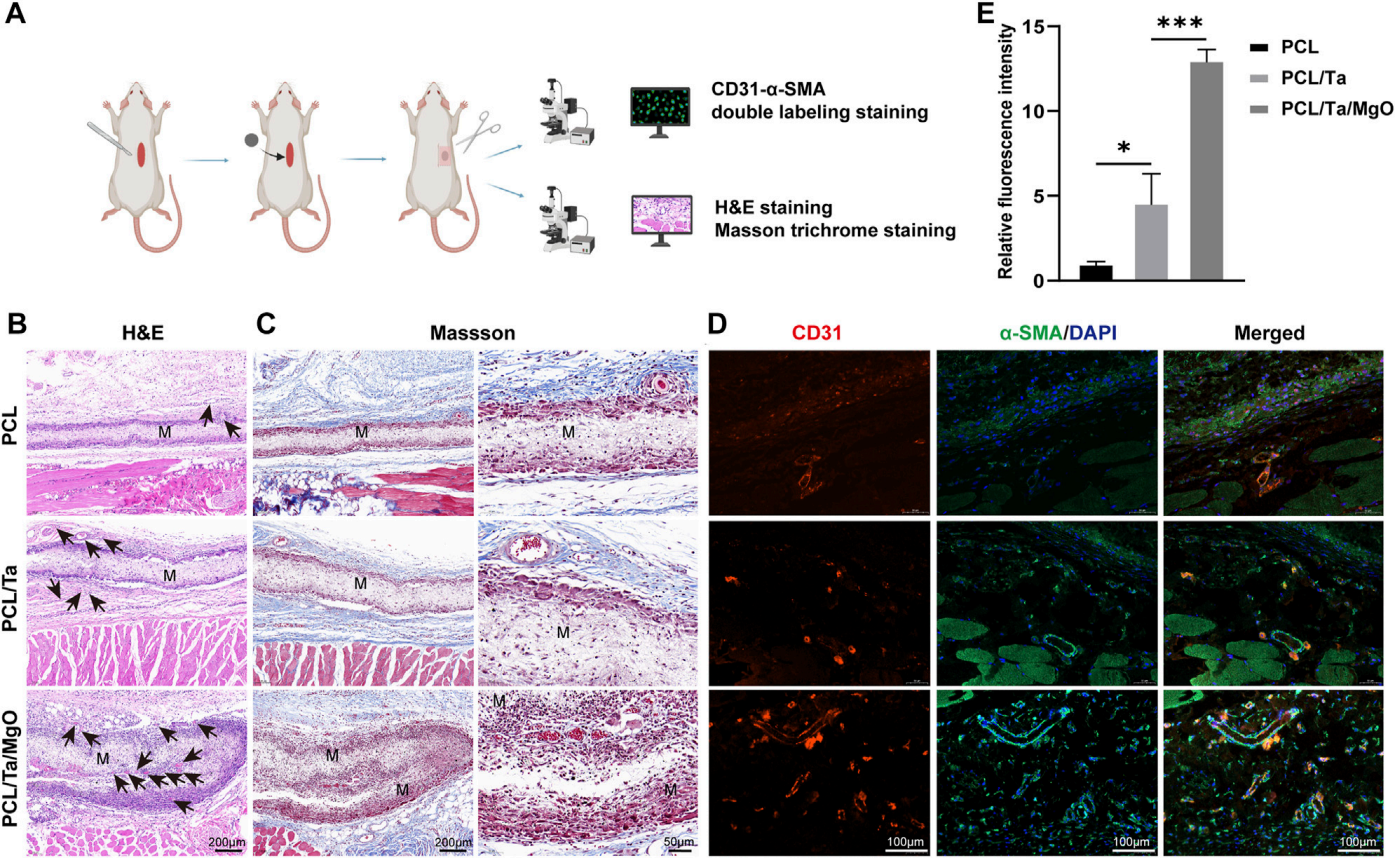
Evaluation of the *in vivo* subcutaneous biocompatibility and angiogenesis of different membranes at day 14 after implantation. **(A)** Schematic draft of subcutaneous implantation test. **(B)** Representative images of H&E staining. Black arrows: blood vessels; M: membranes. **(C)** Representative images of Masson’s trichrome staining and a magnified view of the membrane region. **(D)** Representative images of immunofluorescence staining for CD31 and *a*-SMA. **(E)** Relative fluorescence intensity of CD31. (*n* = 3) (*p < 0.05, **p < 0.01, ***p < 0.001, and ****p < 0.0001).

Animal experiments were performed to verify the ability of the PCL/Ta/MgO periosteum replacement to promote osteogenesis and angiogenesis. Subcutaneous implantation experiments were conducted for the *in vitro* assessment of biocompatibility and vascularization ability (Figure 6A). Samples were obtained 14 days after implantation, and sections containing the implanted membranes were analyzed by H&E and Masson’s trichrome staining. The infiltration of mixed inflammatory cells was observed around the periosteum replacement, including lymphocytes, macrophages, and a few neutrophils (red arrows in Supplementary Figure S2). H&E and Masson’s staining in the pure PCL group showed little neovascularization, while more neovessels were observed in the PCL/Ta and PCL/Ta/MgO groups, with the highest amount of neovascularization observed in the PCL/Ta/MgO group (Figures 6B,C). To clearly display vascular formation, anti-CD31 (biomarker for vascular endothelial cells)-α-SMA (biomarker for smooth muscle cells) immunofluorescence double labeling was performed. In Figure 6D, CD31 (SAF005, AiFang Biological) and *a*-SMA expression appear as red and green fluorescence, respectively. The integrated density of red fluorescence in each section was further quantified. With successive doping of TaNPs and nano-MgO, the neovascularization increased gradually, with the PCL/Ta/MgO group showing the highest number of neovessels. TaNPs and nano-MgO showed a synergistic effect in promoting angiogenesis, consistent with the results of the *in vitro* experiments. Quantification of CD31 immunofluorescence also verified the trend and the highest neovascularization around the PCL/Ta/MgO membrane (Figure 6E). The incorporation of TaNPs significantly promoted angiogenic properties both *in vitro* and *in vivo,* which will be followed up in future studies.

**FIGURE 7:**
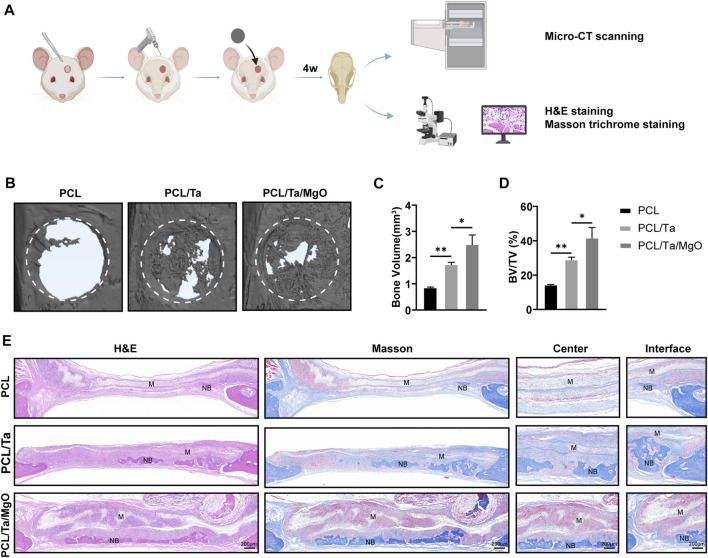
Evaluation of the *in vivo* bone formation in rat skull defects using different membranes. **(A)** Schematic draft of the procedure. **(B)** Representative scanning images by micro-CT showing the reconstructed defect areas and **(C)** newly formed bone volume (BV) and **(D)** BV/TV quantification showed best repairing effects originating from PCL/Ta/MgO membranes (*n* = 3). **(E)** Representative histological images of different membranes with H&E and Masson staining after 4 weeks. (M: material; NB: new bone) (*p < 0.05, **p < 0.01, ***p < 0.001, and ****p < 0.0001).

The critical-sized calvarial defect model was used to evaluate the effects of PCL, PCL/Ta, and PCL/Ta/MgO nanofiber membranes on the promotion of periosteum and bone regeneration (Figure 7A). Four weeks after surgery, skull samples were harvested and scanned by micro-CT. The resulting images were processed for 3D reconstruction (Figure 7B). The PCL/Ta/MgO membrane group showed the best regeneration of the defect. Quantitative analysis of BV and BV/TV showed the same trend (Figures 7C,D). The pure PCL membranes showed the poorest repair effect, with a bone volume fraction of 14.36 ± 0.75%. The PCL/Ta group showed a better performance compared to pure PCL, with a bone fraction volume of 29.07 ± 1.80%. The PCL/Ta/MgO membrane also induced the highest bone formation capacity, with a bone volume fraction of 41.76 ± 6.40%. The new bone volume in defect areas followed the same trend.

The samples were embedded and sections were used for H&E and Masson’s trichrome staining after decalcification to compare the microscopic histological details of the defect area (Figure 7E). The H&E-stained histological sections from both subcutaneous implantation and critical-sized calvarial defect models showed that the PCL/Ta and PCL/Ta/MgO membranes were thicker than the pure PCL membrane and that the PCL/Ta/MgO membrane was the thickest. However, Masson’s trichrome staining revealed an apparent fibrous capsule around the pure PCL membrane on day 14 after implantation; the capsule was thinner around the PCL/Ta membranes and was almost indiscernible around the PCL/Ta/MgO membranes (Supplementary Figure S3). The fibrous capsule was mainly composed of inflammatory cells and dense collagen produced by fibroblasts, which was developed to act against biomaterials by the systemic immune system. The formation of this fibrous capsule around biomaterials is characteristic of a foreign body reaction (FBR), in which a thinner fibrous capsule suggests a lower FBR to biomaterials (Hernandez et al., 2021). In the present study, the fibrous capsule around the PCL membrane was similar to that of many other known biocompatible implants (Higgins et al., 2009; Valentin et al., 2010); therefore, the PCL/Ta and PCL/Ta/MgO membranes appeared to show better biocompatibility at 14 days. However, Liu et al. reported a slightly higher FBR for the PCL/gelatin/MgO membrane compared to PCL/gelatin membrane at 7 d after subcutaneous implantation, which they speculated might be associated with the toxicity of local high concentrations of Mg^2+^ caused by the early-burst release from the membrane, or the combined effect of magnesium hydroxide, the hydrolysate of MgO, and the high concentration of Mg^2+^ (Ge et al., 2011; Mahmoud et al., 2016). However, on day 14, they observed that the inflammatory response to the PCL/gelatin/MgO membrane had subsided, likely due to the decreased number of MgO nanoparticles at the later stage of implantation (Liu M. et al., 2022). Further observation by H&E staining in the present study revealed significantly higher cell infiltration into the PCL/Ta/MgO membrane compared to the PCL and PCL/Ta membranes at day 14. With the incorporation of TaNPs and nano-MgO, more inflammatory cells including lymphocytes, neutrophils, and macrophages infiltrated the membranes (Supplementary Figure S2). Liu et al. also observed higher inflammatory cell infiltration in PCL/gelatin/MgO membranes compared to PCL/gelatin membranes, with similar trends in thickness (Liu M. et al., 2022). Moreover, immunohistochemical staining showed that the infiltrated cells were mainly CD11b-positive cells, including monocytes, neutrophils, natural killer cells, granulocytes, and macrophages (Thevenot et al., 2010; Darling et al., 2020). Combined with previous studies, we hypothesized that the increased thickness of the PCL/Ta and PCL/Ta/MgO membranes was mainly due to the increased infiltration of inflammatory cells in the nanofiber membranes. However, the increase in infiltrated inflammatory cells in the PCL/Ta/MgO membranes did not result in a thicker fibrous capsule compared to that in the PCL membranes after 14 d in the subcutaneous implantation model and 4 weeks in the critical-sized calvarial defect model, and instead promoted neovascularization and bone regeneration. Therefore, inflammatory cell infiltration in the PCL/Ta and PCL/Ta/MgO membranes may create a favorable immune microenvironment to facilitate vascularization and tissue repair.

**FIGURE 8:**
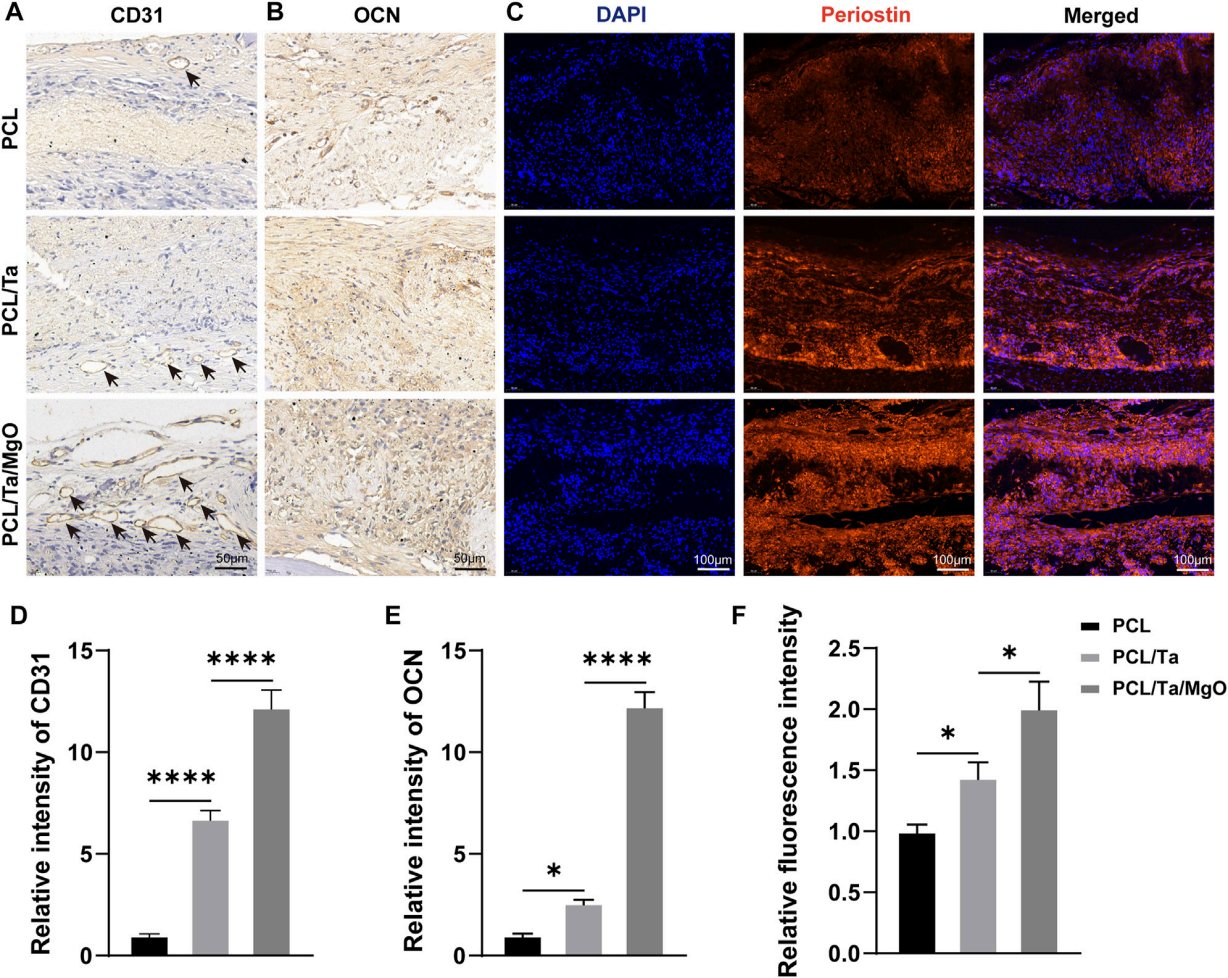
Representative images of immunohistochemical staining for **(A)** CD31 and **(B)** OCN. Black arrows: vessels around the membranes. **(C)** Immunofluorescence staining of periostin in the defect areas at 4 weeks. Quantitative assessment of the staining intensities of **(D)** CD31, **(E)** OCN, and **(F)** periostin (*n* = 3). (*p < 0.05, **p < 0.01, ***p < 0.001, and ****p < 0.0001).

Furthermore, the defect area showed limited repair in the PCL group, while the bone defect areas in the PCL/Ta and PCL/Ta/MgO groups shrank obviously compared to that of PCL. Masson’s staining of the collagen fibers demonstrated sparse fibers in bone defect areas in the PCL group, with higher abundance in both groups doped with metal nanoparticles. In terms of bone formation in the defect areas, little new bone tissue was observed in the defect area of the pure PCL group. The incorporation of TaNPs promoted remarkable new bone formation, which was related to its confirmed robust osteogenic ability. The favorable immune regulation might have also contributed to facilitating bone formation. The PCL/Ta/MgO group showed the highest amount of newly formed bone, and the defect field was almost completely filled. Immunohistochemical staining for CD31 (angiogenic biomarker) and OCN (osteogenic biomarker) of the sliced sections (Figures 8A,B) showed that the numbers of OCN-positive and CD31-positive cells increased with the integration of TaNPs and nano-MgO, thus confirming the prominent osteogenic and angiogenic properties *in vivo* of the PCL/Ta/MgO periosteum replacement. Quantification of the staining intensities of the two markers (Figures 8D,E) confirmed the staining results.

Periostin, originally termed osteoblast-specific factor 2 (OSF-2) because of its localization to the periodontal ligament and periosteum, is a matricellular protein member of the fasciclin family (Nikoloudaki et al., 2020). The constitutive expression of matricellular proteins in adult tissues is restricted but is increased during embryonic development and repair after injury (Duchamp de Lageneste and Colnot, 2019). Periostin is specifically expressed in collagen-dense areas of connective tissue in physiological conditions, including the periodontal ligament, periosteum, and cardiac valve, and is normally highly expressed in the cambial layer of the activated periosteum. It is also closely associated with tissue regeneration post-injury (Kii, 2019). Periostin is involved in extracellular matrix stabilization, including collagen fibrillogenesis and cross-linking (Nikoloudaki et al., 2020). Oriane et al. reported overexpression of periostin genes in periosteum cells in response to bone injury as well as periostin expression in the callus and osteoblasts within new bone trabeculae (Duchamp de Lageneste et al., 2018). Following a bone defect, periostin plays important roles through all stages of bone regeneration, from the initial activation of stem cells in the periosteum, the active soft and hard callus phase, and the final phase of bone bridging (Duchamp de Lageneste and Colnot, 2019). Previous studies used periostin as a biomarker to examine periosteum repair (Duchamp de Lageneste et al., 2018; Wan et al., 2022). As shown in Figure 8C, periostin was stained as red fluorescence, the intensity of which was higher in the PCL/Ta and PCL/Ta/MgO groups. The results also demonstrated the highest periostin expression levels in the periosteum of the PCL/Ta/MgO group. The relative fluorescence intensity (Figure 8F) compared to the pure PCL group showed a more visualized effect on promoting periosteum regeneration.

The observed bone formation and neovascularization *in vivo* were both consistent with the *in vitro* experiments and radiographical results. Combined with the periostin expression findings, these results further demonstrated the excellent potential of the PCL/Ta/MgO membrane to provide periosteum regeneration by employing the advantages of TaNPs and nano-MgO to achieve more synergetic effects on bone regeneration. Overall, the PCL/Ta/MgO periosteum replacement showed the best performance in inducing bone regeneration in critical-sized bone defects.

## Conclusion

Overall, the PCL-based electrospinning periosteum replacement containing TaNPs and nano-MgO prepared in this study showed an osteogenic–angiogenic coupling effect. The PCL/Ta/MgO periosteum replacement exhibited good biocompatibility *in vitro* and *in vivo*. The presence of TaNPs and the sustained release of Mg^2+^ had an inductive effect on the osteogenic differentiation of BMSCs and an angiogenic effect on EPCs. Moreover, the results of subcutaneous implantation confirmed the enhanced angiogenic ability, while the repair of critical-sized calvarial defects verified its excellent osteogenic ability and improved periosteum regeneration. Combined, these results demonstrated the promising potential of the PCL/Ta/MgO membrane with a coupling effect as a periosteum replacement Boyan et al., 2017.

## Data Availability

The raw data supporting the conclusions of this manuscript will be made available by the authors, without undue reservation, to any qualified researcher.

## References

[B1] Allen M. R. Hock J. M. Burr D. B. (2004). Periosteum: Biology, regulation, and response to osteoporosis therapies. Bone 35 (5), 1003–1012. 10.1016/j.bone.2004.07.014 15542024

[B2] An S. Ling J. Gao Y. Xiao Y. (2012). Effects of varied ionic calcium and phosphate on the proliferation, osteogenic differentiation and mineralization of human periodontal ligament cells *in vitro* . J. Periodontal Res. 47 (3), 374–382. 10.1111/j.1600-0765.2011.01443.x 22136426

[B3] Bombaldi de Souza R. F. Bombaldi de Souza F. C. Thorpe A. Mantovani D. Popat K. C. Moraes Â M. (2020). Phosphorylation of chitosan to improve osteoinduction of chitosan/xanthan-based scaffolds for periosteal tissue engineering. Int. J. Biol. Macromol. 143, 619–632. 10.1016/j.ijbiomac.2019.12.004 31811849

[B4] Boyan B. D. Lotz E. M. Schwartz Z. (2017). Roughness and hydrophilicity as osteogenic biomimetic surface properties. Tissue Eng. Part A 23 (24), 1479–1489. 10.1089/ten.TEA.2017.0048 28793839 PMC5729880

[B5] Calori G. M. Mazza E. Colombo M. Ripamonti C. (2011). The use of bone-graft substitutes in large bone defects: Any specific needs? Injury 42 (2), S56–S63. 10.1016/j.injury.2011.06.011 21752369

[B6] Chen J. X. Lawrence M. L. Cunningham G. Christman B. W. Meyrick B. (2004). HSP90 and Akt modulate Ang-1-induced angiogenesis via NO in coronary artery endothelium. J. Appl. Physiol. (1985). 96 (2), 612–620. 10.1152/japplphysiol.00728.2003 14555685

[B7] Chen K. Lin X. Zhang Q. Ni J. Li J. Xiao J. (2015). Decellularized periosteum as a potential biologic scaffold for bone tissue engineering. Acta Biomater. 19, 46–55. 10.1016/j.actbio.2015.02.020 25725472

[B8] Chen Y. Sheng W. Lin J. Fang C. Deng J. Zhang P. (2022). Magnesium oxide nanoparticle coordinated phosphate-functionalized chitosan injectable hydrogel for osteogenesis and angiogenesis in bone regeneration. ACS Appl. Mat. Interfaces 14 (6), 7592–7608. 10.1021/acsami.1c21260 35119809

[B9] Darling N. J. Xi W. Sideris E. Anderson A. R. Pong C. Carmichael S. T. (2020). Click by click microporous annealed particle (MAP) scaffolds. Adv. Healthc. Mat. 9 (10), e1901391. 10.1002/adhm.201901391 PMC7340246 32329234

[B10] Duchamp de Lageneste O. Colnot C. (2019). Periostin in bone regeneration. Adv. Exp. Med. Biol. 1132, 49–61. 10.1007/978-981-13-6657-4_6 31037624

[B11] Duchamp de Lageneste O. Julien A. Abou-Khalil R. Frangi G. Carvalho C. Cagnard N. (2018). Periosteum contains skeletal stem cells with high bone regenerative potential controlled by Periostin. Nat. Commun. 9 (1), 773. 10.1038/s41467-018-03124-z 29472541 PMC5823889

[B12] Dwek J. R. (2010). The periosteum: What is it, where is it, and what mimics it in its absence? Skelet. Radiol. 39 (4), 319–323. 10.1007/s00256-009-0849-9 PMC2826636 20049593

[B13] Facchetti D. Hempel U. Martocq L. Smith A. M. Koptyug A. Surmenev R. A. (2021). Heparin enriched-WPI coating on Ti6Al4V increases hydrophilicity and improves proliferation and differentiation of human bone marrow stromal cells. Int. J. Mol. Sci. 23 (1), 139. 10.3390/ijms23010139 35008562 PMC8745389

[B14] Felice B. Sánchez M. A. Socci M. C. Sappia L. D. Gómez M. I. Cruz M. K. (2018). Controlled degradability of PCL-ZnO nanofibrous scaffolds for bone tissue engineering and their antibacterial activity. Mater. Sci. Eng. C 93, 724–738. 10.1016/j.msec.2018.08.009 30274106

[B15] Franceschi R. T. Xiao G. Jiang D. Gopalakrishnan R. Yang S. Reith E. (2003). Multiple signaling pathways converge on the Cbfa1/Runx2 transcription factor to regulate osteoblast differentiation. Connect. Tissue Res. 44 (1), 109–116. 10.1080/03008200390152188 PMC3564252 12952183

[B16] Gao B. Deng R. Chai Y. Chen H. Hu B. Wang X. (2019). Macrophage-lineage TRAP+ cells recruit periosteum-derived cells for periosteal osteogenesis and regeneration. J. Clin. Invest. 129 (6), 2578–2594. 10.1172/jci98857 30946695 PMC6538344

[B17] Ge S. Wang G. Shen Y. Zhang Q. Jia D. Wang H. (2011). Cytotoxic effects of MgO nanoparticles on human umbilical vein endothelial cells *in vitro* . IET Nanobiotechnol. 5 (2), 36–40. 10.1049/iet-nbt.2010.0022 21495778

[B18] Hernandez J. L. Park J. Yao S. Blakney A. K. Nguyen H. V. Katz B. H. (2021). Effect of tissue microenvironment on fibrous capsule formation to biomaterial-coated implants. Biomaterials 273, 120806. 10.1016/j.biomaterials.2021.120806 33905960 PMC8135119

[B19] Higgins D. M. Basaraba R. J. Hohnbaum A. C. Lee E. J. Grainger D. W. Gonzalez-Juarrero M. (2009). Localized immunosuppressive environment in the foreign body response to implanted biomaterials. Am. J. Pathol. 175 (1), 161–170. 10.2353/ajpath.2009.080962 19528351 PMC2708803

[B20] Ho-Shui-Ling A. Bolander J. Rustom L. E. Johnson A. W. Luyten F. P. Picart C. (2018). Bone regeneration strategies: Engineered scaffolds, bioactive molecules and stem cells current stage and future perspectives. Biomaterials 180, 143–162. 10.1016/j.biomaterials.2018.07.017 30036727 PMC6710094

[B21] Ji C. Bi L. Li J. Fan J. (2019). Salvianolic acid B-loaded chitosan/hydroxyapatite scaffolds promotes the repair of segmental bone defect by angiogenesis and osteogenesis Int. J. Nanomedicine 14, 8271–8284. 10.2147/ijn.S219105 31686820 PMC6800558

[B22] Kang C. Wei L. Song B. Chen L. Liu J. Deng B. (2017). Involvement of autophagy in tantalum nanoparticle-induced osteoblast proliferation. Int. J. Nanomedicine 12, 4323–4333. 10.2147/ijn.S136281 28652735 PMC5473603

[B23] Khorsand B. Elangovan S. Hong L. Kormann M. S. D. Salem A. K. (2019). A bioactive collagen membrane that enhances bone regeneration. J. Biomed. Mat. Res. 107 (6), 1824–1832. 10.1002/jbm.b.34275 PMC6531367 30466196

[B24] Kii I. (2019). Practical application of periostin as a biomarker for pathological conditions. Adv. Exp. Med. Biol. 1132, 195–204. 10.1007/978-981-13-6657-4_18 31037636

[B25] Kim H. D. Jang H. L. Ahn H. Y. Lee H. K. Park J. Lee E. S. (2017). Biomimetic whitlockite inorganic nanoparticles-mediated *in situ* remodeling and rapid bone regeneration. Biomaterials 112, 31–43. 10.1016/j.biomaterials.2016.10.009 27744219

[B26] Lai Y. Li Y. Cao H. Long J. Wang X. Li L. (2019). Osteogenic magnesium incorporated into PLGA/TCP porous scaffold by 3D printing for repairing challenging bone defect. Biomaterials 197, 207–219. 10.1016/j.biomaterials.2019.01.013 30660996

[B27] Levine B. R. Sporer S. Poggie R. A. Della Valle C. J. Jacobs J. J. (2006). Experimental and clinical performance of porous tantalum in orthopedic surgery. Biomaterials 27 (27), 4671–4681. 10.1016/j.biomaterials.2006.04.041 16737737

[B28] Lin Z. Shen D. Zhou W. Zheng Y. Kong T. Liu X. (2021). Regulation of extracellular bioactive cations in bone tissue microenvironment induces favorable osteoimmune conditions to accelerate *in situ* bone regeneration. Bioact. Mat. 6 (8), 2315–2330. 10.1016/j.bioactmat.2021.01.018 PMC7840811 33553818

[B29] Liu M. Wang R. Liu J. Zhang W. Liu Z. Lou X. (2022a). Incorporation of magnesium oxide nanoparticles into electrospun membranes improves pro-angiogenic activity and promotes diabetic wound healing. Biomater. Adv. 133, 112609. 10.1016/j.msec.2021.112609 35525752

[B30] Liu W. B. Ma M. S. Lei Z. H. Xiong Z. X. Tao T. H. Lei P. F. (2022b). Intra-articular injectable hydroxypropyl chitin/hyaluronic acid hydrogel as bio-lubricant to attenuate osteoarthritis progression. Mater. Des. 217, 110579. 10.1016/j.matdes.2022.110579

[B31] Lu Z. Wang W. Zhang J. Bártolo P. Gong H. Li J. (2020). Electrospun highly porous poly(L-lactic acid)-dopamine-SiO(2) fibrous membrane for bone regeneration. Mater. Sci. Eng. C 117, 111359. 10.1016/j.msec.2020.111359 32919696

[B32] Ma L. Cheng S. Ji X. Zhou Y. Zhang Y. Li Q. (2020). Immobilizing magnesium ions on 3D printed porous tantalum scaffolds with polydopamine for improved vascularization and osteogenesis. Mater. Sci. Eng. C 117, 111303. 10.1016/j.msec.2020.111303 32919664

[B33] Mahmoud A. Ezgi Ö. Merve A. Özhan G. (2016). *In vitro* toxicological assessment of magnesium oxide nanoparticle exposure in several mammalian cell types. Int. J. Toxicol. 35 (4), 429–437. 10.1177/1091581816648624 27177543

[B34] Mei S. Yang L. Pan Y. Wang D. Wang X. Tang T. (2019). Influences of tantalum pentoxide and surface coarsening on surface roughness, hydrophilicity, surface energy, protein adsorption and cell responses to PEEK based biocomposite. Colloids Surfaces B Biointerfaces 174, 207–215. 10.1016/j.colsurfb.2018.10.081 30465995

[B35] Nikoloudaki G. Creber K. Hamilton D. W. (2020). Wound healing and fibrosis: A contrasting role for periostin in skin and the oral mucosa. Am. J. Physiology-Cell Physiology 318 (6), C1065–c1077. 10.1152/ajpcell.00035.2020 PMC7311745 32267719

[B36] Orwoll E. S. (2003). Toward an expanded understanding of the role of the periosteum in skeletal health. J. Bone Min. Res. 18 (6), 949–954. 10.1359/jbmr.2003.18.6.949 12817746

[B37] Park C. Seong Y. J. Kang I. G. Song E. H. Lee H. Kim J. (2019). Enhanced osseointegration ability of poly(lactic acid) via tantalum sputtering-based plasma immersion ion implantation. ACS Appl. Mat. Interfaces 11 (11), 10492–10504. 10.1021/acsami.8b21363 30802030

[B38] Park J. Zobaer T. Sutradhar A. (2021). A two-scale multi-resolution topologically optimized multi-material design of 3D printed craniofacial bone implants. Micromachines (Basel) 12 (2), 101. 10.3390/mi12020101 33498498 PMC7909579

[B39] Qian H. Lei T. Lei P. Hu Y. (2021). Additively manufactured tantalum implants for repairing bone defects: A systematic review. Tissue Eng. Part B Rev. 27 (2), 166–180. 10.1089/ten.TEB.2020.0134 32799765

[B40] Qiu P. Li M. Chen K. Fang B. Chen P. Tang Z. (2020a). Periosteal matrix-derived hydrogel promotes bone repair through an early immune regulation coupled with enhanced angio- and osteogenesis. Biomaterials 227, 119552. 10.1016/j.biomaterials.2019.119552 31670079

[B41] Qiu W. X. Ma X. L. Lin X. Zhao F. Li D. J. Chen Z. H. (2020b). Deficiency of Macf1 in osterix expressing cells decreases bone formation by Bmp2/Smad/Runx2 pathway. J. Cell. Mol. Med. 24 (1), 317–327. 10.1111/jcmm.14729 31709715 PMC6933318

[B42] Shi X. Fujie T. Saito A. Takeoka S. Hou Y. Shu Y. (2014). Periosteum-mimetic structures made from freestanding microgrooved nanosheets. Adv. Mat. 26 (20), 3290–3296. 10.1002/adma.201305804 24616147

[B43] Shuai Y. Lu H. Lv R. Wang J. Wan Q. Mao C. (2021). Biomineralization directed by prenucleated calcium and phosphorus nanoclusters improving mechanical properties and osteogenic potential of *Antheraea pernyi* silk fibroin‐based artificial periosteum. Adv. Healthc. Mat. 10 (8), e2001695. 10.1002/adhm.202001695 33720549

[B44] Sun Y. Liu T. Hu H. Xiong Z. Zhang K. He X. (2022). Differential effect of tantalum nanoparticles versus tantalum micron particles on immune regulation. Mater. Today Bio 16, 100340. 10.1016/j.mtbio.2022.100340 PMC9278074 35847379

[B45] Thevenot P. T. Nair A. M. Shen J. Lotfi P. Ko C. Y. Tang L. (2010). The effect of incorporation of SDF-1α into PLGA scaffolds on stem cell recruitment and the inflammatory response. Biomaterials 31 (14), 3997–4008. 10.1016/j.biomaterials.2010.01.144 20185171 PMC2838969

[B46] Toffoli A. Parisi L. Bianchi M. G. Lumetti S. Bussolati O. Macaluso G. M. (2020). Thermal treatment to increase titanium wettability induces selective proteins adsorption from blood serum thus affecting osteoblasts adhesion. Mater. Sci. Eng. C 107, 110250. 10.1016/j.msec.2019.110250 31761226

[B47] Torstrick F. B. Evans N. T. Stevens H. Y. Gall K. Guldberg R. E. (2016). Do surface porosity and pore size influence mechanical properties and cellular response to PEEK? Clin. Orthop. Relat. Res. 474 (11), 2373–2383. 10.1007/s11999-016-4833-0 27154533 PMC5052186

[B48] Valentin J. E. Turner N. J. Gilbert T. W. Badylak S. F. (2010). Functional skeletal muscle formation with a biologic scaffold. Biomaterials 31 (29), 7475–7484. 10.1016/j.biomaterials.2010.06.039 20638716 PMC2922042

[B49] Wan Q. Q. Jiao K. Ma Y. X. Gao B. Mu Z. Wang Y. R. (2022). Smart, biomimetic periosteum created from the cerium(III, IV) oxide-mineralized eggshell membrane. ACS Appl. Mat. Interfaces 14 (12), 14103–14119. 10.1021/acsami.2c02079 35306805

[B50] Xiong Z. X. Liu W. B. Qian H. Lei T. He X. Hu Y. H. (2021). Tantalum nanoparticles reinforced PCL scaffolds using direct 3D printing for bone tissue engineering. Front. Mat. 8. 10.3389/fmats.2021.609779

[B51] Yuan Z. Wan Z. Wei P. Lu X. Mao J. Cai Q. (2020). Dual-Controlled release of icariin/Mg(2+) from biodegradable microspheres and their synergistic upregulation effect on bone regeneration. Adv. Healthc. Mat. 9 (11), e2000211. 10.1002/adhm.202000211 32338458

[B52] Yuan Z. Wei P. Huang Y. Zhang W. Chen F. Zhang X. (2019). Injectable PLGA microspheres with tunable magnesium ion release for promoting bone regeneration. Acta Biomater. 85, 294–309. 10.1016/j.actbio.2018.12.017 30553873

[B53] Zeng Y. Hoque J. Varghese S. (2019). Biomaterial-assisted local and systemic delivery of bioactive agents for bone repair. Acta Biomater. 93, 152–168. 10.1016/j.actbio.2019.01.060 30711659 PMC6615988

[B54] Zhai W. Lu H. Wu C. Chen L. Lin X. Naoki K. (2013). Stimulatory effects of the ionic products from Ca-Mg-Si bioceramics on both osteogenesis and angiogenesis *in vitro* . Acta Biomater. 9 (8), 8004–8014. 10.1016/j.actbio.2013.04.024 23619289

[B55] Zhang C. N. Zhou L. Y. Qian S. J. Gu Y. X. Shi J. Y. Lai H. C. (2021a). Improved response of human gingival fibroblasts to titanium coated with micro-/nano-structured tantalum. Int. J. Implant Dent. 7 (1), 36. 10.1186/s40729-021-00316-z 33937945 PMC8089072

[B56] Zhang G. Liu W. Wang R. Zhang Y. Chen L. Chen A. (2020). The role of tantalum nanoparticles in bone regeneration involves the BMP2/smad4/runx2 signaling pathway Int. J. Nanomedicine 15, 2419–2435. 10.2147/ijn.S245174 32368035 PMC7174976

[B57] Zhang J. Tang L. Qi H. Zhao Q. Liu Y. Zhang Y. (2019). Dual function of magnesium in bone biomineralization. Adv. Healthc. Mat. 8 (21), e1901030. 10.1002/adhm.201901030 31583846

[B58] Zhang X. Liu W. Liu J. Hu Y. Dai H. (2021b). Poly-ε-caprolactone/Whitlockite electrospun bionic membrane with an osteogenic-angiogenic coupling effect for periosteal regeneration. ACS Biomater. Sci. Eng. 7 (7), 3321–3331. 10.1021/acsbiomaterials.1c00426 34148343

[B59] Zhu H. Ji X. Guan H. Zhao L. Zhao L. Liu C. (2019). Tantalum nanoparticles reinforced polyetheretherketone shows enhanced bone formation. Mater. Sci. Eng. C 101, 232–242. 10.1016/j.msec.2019.03.091 31029316

