# Supplementary material for: Tantalum and magnesium nanoparticles enhance the biomimetic properties and osteo-angiogenic effects of PCL membranes
Source: Front Bioeng Biotechnol. 2022 Nov 24;10:1038250. doi: 10.3389/fbioe.2022.1038250 (PMC9730409; doi:10.3389/fbioe.2022.1038250)
Supplement: Supplementary file 1 [file Table1.DOCX]

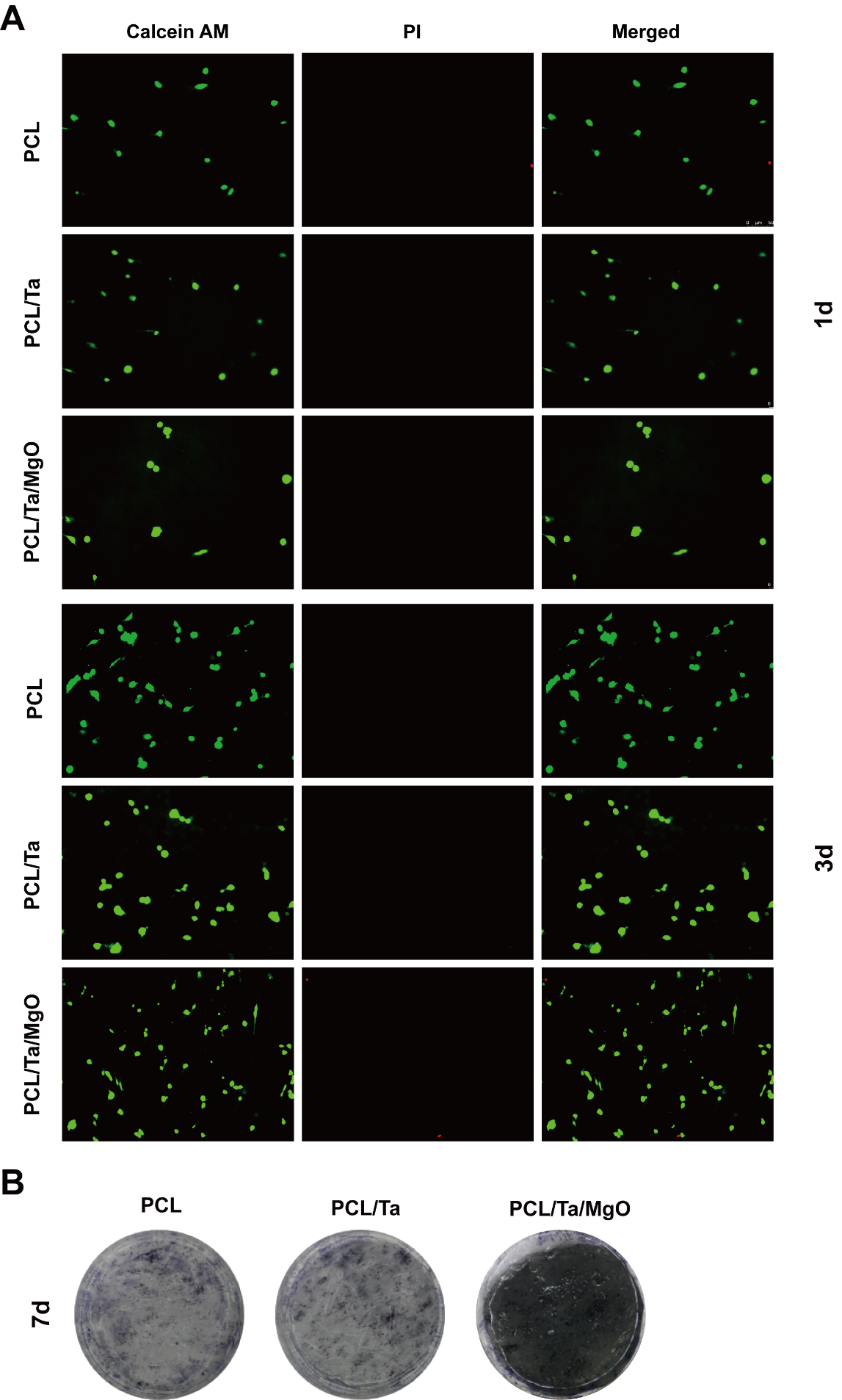


**Figure S1.** (A) Live/Dead staining of EPCs cultured on the membranes after 1 d and 3 d; (B) Representative images of ALP staining at day 7 after osteogenic induction.

**Figure S2.**
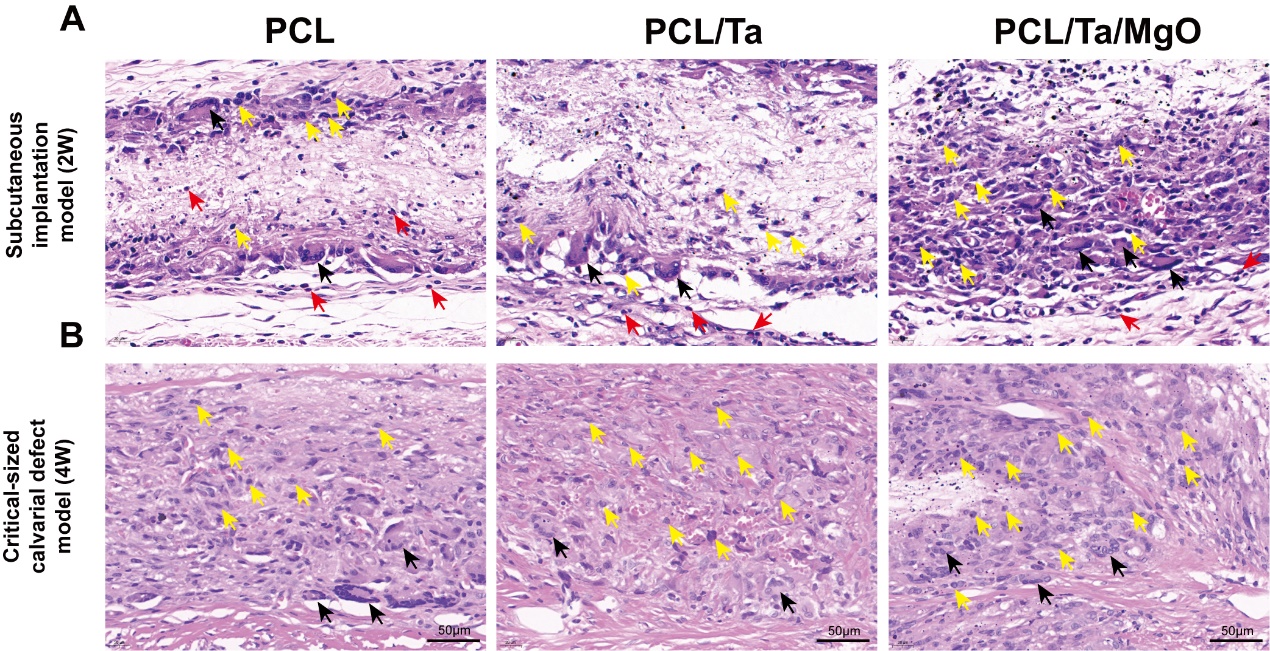
Representative images of inflammatory cells infiltrated in the membranes from (A) subcutaneous implantation model at 2 weeks and (B) critical-sized calvarial defect model at 4weeks. Red arrows: neutrophils; black arrows: macrophages; yellow arrows: lymphocytes.


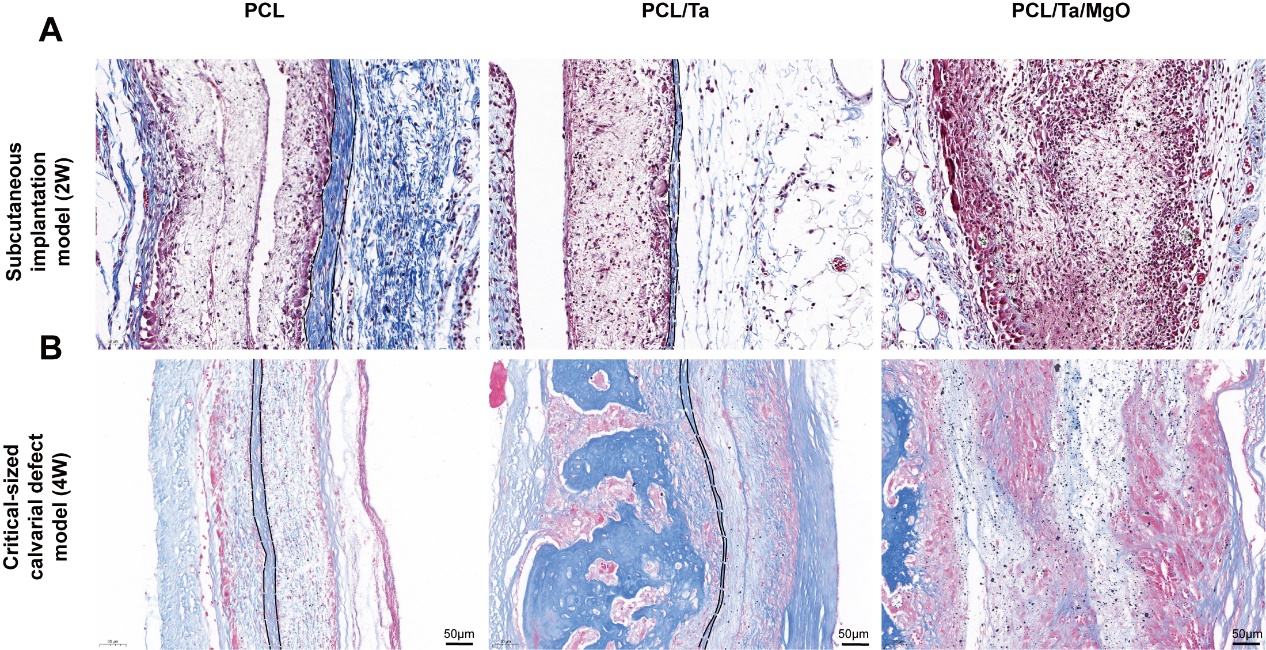


**Figure S3.** Representative images of fibrous capsule formation around membranes from (A) subcutaneous implantation model at 2 weeks and (B) critical-sized calvarial defect model at 4weeks. The black dashed line marks fibrous capsule.


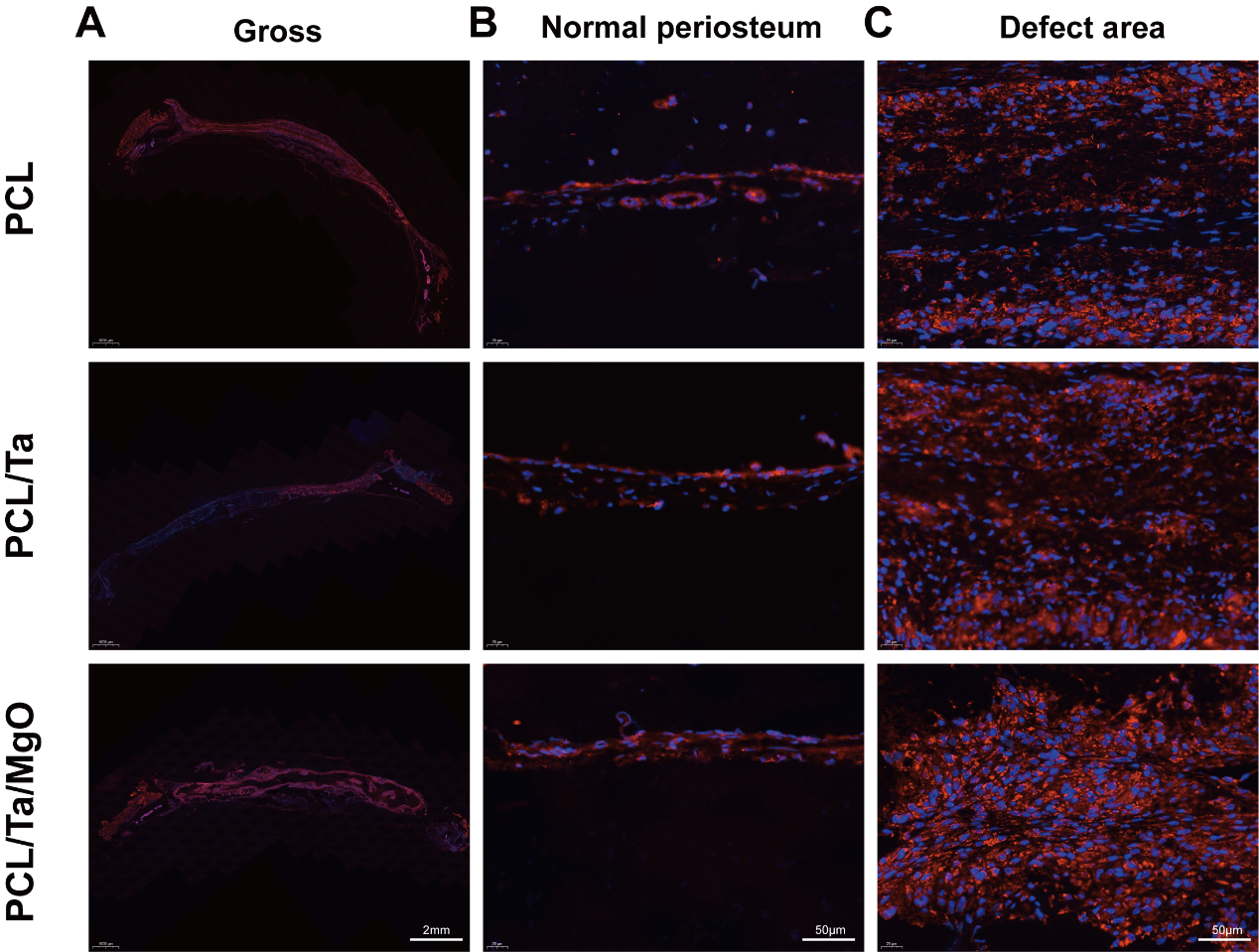


**Figure S4.** Immunofluorescence staining of periostin and DAPI in normal periosteum and defect areas at 4 weeks after surgery. (A) Gross view of the slices; (B) Expression of periostin in the normal periosteum. (C) Expression of periostin in the defect areas.
